# Normalization of γ-glutamyl transferase levels is associated with better metabolic control in individuals with nonalcoholic fatty liver disease

**DOI:** 10.1186/s12876-021-01790-w

**Published:** 2021-05-10

**Authors:** Qianqian Ma, Xianhua Liao, Congxiang Shao, Yansong Lin, Tingfeng Wu, Yanhong Sun, Shi-Ting Feng, Junzhao Ye, Bihui Zhong

**Affiliations:** 1 Department of Gastroenterology, The First Affiliated Hospital of Sun Yat-Sen University, No. 58 Zhongshan II Road, Yuexiu District, Guangzhou, 510080 China; 2 Department of Clinical Laboratories, The First Affiliated Hospital of Sun Yat-Sen University, Guangzhou, China; 3 Department of Radiology, The First Affiliated Hospital of Sun Yat-Sen University, Guangzhou, China

**Keywords:** Gamma glutamyl transferase, Nonalcoholic fatty liver disease, Liver function, Metabolic syndrome

## Abstract

**Background:**

The normalization of liver biochemical parameters usually reflects the histological response to treatment for nonalcoholic fatty liver disease (NAFLD). Researchers have not clearly determined whether different liver enzymes exhibit various metabolic changes during the follow-up period in patients with NAFLD.

**Methods:**

We performed a retrospective analysis of patients with NAFLD who were receiving therapy from January 2011 to December 2019. Metabolism indexes, including glucose levels, lipid profiles, uric acid levels and liver biochemical parameters, were measured. Magnetic resonance imaging-based proton density fat fraction (MRI-PDFF) and liver ultrasound were used to evaluate steatosis. All patients received recommendations for lifestyle modifications and guideline-recommended pharmacological treatments with indications for drug therapy for metabolic abnormalities.

**Results:**

Overall, 1048 patients with NAFLD were included and received lifestyle modification recommendations and pharmaceutical interventions, including 637 (60.7%) patients with abnormal GGT levels and 767 (73.2%) patients with abnormal ALT levels. Patients with concurrent ALT and GGT abnormalities presented higher levels of metabolism indexes and higher liver fat content than those in patients with single or no abnormalities. After 12 months of follow-up, the cumulative normalization rate of GGT was considerably lower than that of ALT (38% vs. 62%, *P* < 0.001). Greater weight loss resulted in higher cumulative normalization rates of GGT and ALT. Weight loss (OR = 1.21, 95% CI 1.11–1.32, *P* < 0.001), ALT normalization (OR = 2.75, 95% CI 1.41–5.36, *P* = 0.01) and lower TG and HOMA-IR values (OR = 2.03, 95% CI 1.11–3.71, *P* = 0.02; OR = 2.04, 95% CI 1.07–3.89, *P* = 0.03) were independent protective factors for GGT normalization. Elevated baseline GGT (OR = 0.99, 95% CI 0.98–0.99, *P* = 0.01) was a risk factor.

**Conclusions:**

For NAFLD patients with concurrently increased ALT and GGT levels, a lower normalization rate of GGT was observed, rather than ALT. Good control of weight and insulin resistance was a reliable predictor of GGT normalization.

**Supplementary Information:**

The online version contains supplementary material available at 10.1186/s12876-021-01790-w.

## Background

Nonalcoholic fatty liver disease (NAFLD), recently renamed metabolic associated fatty liver disease (MAFLD) with positive diagnostic criteria, rather than exclusion of secondary causes of steatosis, is a clinical syndrome characterized by excess lipid storage in hepatocytes and has been acknowledged as the most common chronic liver disease worldwide [1–3]. The incidence of NAFLD continues to increase rapidly, with an estimated 3.6 million patients diagnosed with NAFLD annually based on its global prevalence of up to 25% [4]. In addition to the progression of NAFLD from steatosis to steatohepatitis, fibrosis, cirrhosis, liver failure and carcinoma [5], NAFLD promotes extrahepatic metabolic disturbances, including hypertension, hyperuricemia, hyperlipemia, hyperglycemia, and eventually contributes to a poor prognosis, leading to cardiovascular disease, type 2 diabetes and other metabolic compilations [6]. Therefore, clinical parameters associated with the remission of metabolic abnormalities must be identified when monitoring the effectiveness of treatments for NAFLD.

The liver enzymes alanine aminotransferase (ALT) and γ-glutamyl transferase (GGT) are routine clinical biochemical markers of injured liver cells that are applied to screen for NAFLD or nonalcoholic hepatitis [7]. The primary physical location of ALT is the cytoplasm of liver cells, and its increased level in serum often indicates release from the liver due to cell death. GGT, which is stored in bile duct epithelial cells and hepatocyte microsomes, has long been regarded as a marker of hepatobiliary disease, drug-related liver injuries and excess alcohol consumption [8]. Based on data from emerging studies, both elevated baseline serum ALT and GGT levels are significantly associated with insulin resistance, other metabolic syndromes and an increased risk of long-term complications of myocardial infarction and stroke [9, 10]. A recent study from the TONIC trial reported that decreased serum ALT and GGT levels are associated with improvements in liver histology [11, 12]. However, the relationship between the dynamic changes in GGT and ALT levels and their metabolic treatment responses during the treatment of patients with NAFLD remains to be elucidated [7]. This issue is of particular clinical importance, as these biochemical markers of hepatitis remission may exhibit inconsistent levels during therapy— namely, the levels of one of the markers can decrease to its normal range while the levels of other markers remain abnormal. Relevant studies may be helpful to interpret the results of biochemical assessments conducted during the disease course [6, 7].

In the present study, we aimed to explore the associations of decreased ALT and GGT levels and improvements in metabolic disturbances during the routine treatment of NAFLD over time. Furthermore, we compared the characteristics of patients with NAFLD who presented inconsistent decreases in ALT and GGT levels.

## Methods

### Study design and patients

This single-center retrospective cohort study was conducted in the NAFLD clinic of the First Affiliated Hospital of Sun Yat-sen University, China, from January 1, 2011 to December 31, 2019. The clinical research ethics committee of the First Affiliated Hospital of Sun Yat-sen University approved the research plan, and all subjects provided written informed consent. We extracted individual-level admission data of patients continuously admitted with NAFLD, and the inclusion criteria were as follows: (1) patients aged greater than 18 years; (2) patients with complete anthropometric parameters, laboratory test results and abdominal liver ultrasonography; and (3) an established diagnosis of NAFLD. The diagnosis of NAFLD was defined as (1) liver imaging such as abdominal ultrasonography manifesting steatosis; (2) no drinking history or previous history of alcohol consumption < 30 g/day in males or < 20 g/day in females; and (3) no history of drug-induced liver disease, total parenteral nutrition, hepatolenticular degeneration, autoimmune hepatitis and other specific diseases that may lead to fatty liver [12]. The exclusion criteria included (1) pregnant and breastfeeding women; (2) patients with the specific occupations of athlete or chemical worker; (3) patients with a concomitant malignant tumor or other severe diseases with organ dysfunction; and (4) patients with preexisting cardiovascular diseases or stroke.

### Clinical estimations

Subjects’ information was collected by administering structured questionnaires that included information about basic demographic characteristics (age and sex), previous diseases (hypertension and diabetes), medications, and nicotine and alcohol consumption history. Height, weight, blood pressure, waist circumference and hip circumference were measured by experienced doctors. Blood pressure was measured in the right upper arm with an automatic electronic sphygmomanometer after the patient had rested for more than 15 min, and the average value from 3 successive measurements was recorded [12]. Body mass index (BMI) was calculated as the weight (kg) divided by the square of height (m) [13]. The waist-to-hip ratio (WHR) was calculated as the waist circumference divided by the hip circumference (cm/cm) [13].

### Laboratory measurements

Venous blood samples were collected after a fast for at least 8 h for measurements. Liver biochemical and metabolic parameters, including ALT, aspartate aminotransferase cellulase (AST), GGT, lactate dehydrogenase, choline esterase, leucine arylamidase, glutamate dehydrogenase, direct bilirubin (DBil), total bilirubin (TBil), total bile acid (TBA), lipid profile, fasting blood glucose (FBG), fasting insulin (FINS) and uric acid (UA) levels, were measured. The levels of liver enzymes were measured with the enzymatic-colorimetric method using a conventional automated analyzer (Biochemical analyzer from beckman coulter, Au 5800 System), and the cut-off values for ALT levels were set to 30 U/L for men and 19 U/L for women [14], while the cut-off values for GGT levels were set to 50 U/L for men and women [15]. The homeostatic model assessment of insulin resistance (HOMA-IR) was calculated as follows: FINS (μU/mL) × FBG (mmol/L)/22.5 [16]. A cut-off value of 2.69 was utilized to define insulin resistance (IR) [16]. Fibrosis-4 (FIB-4) index is a simple noninvasive test for liver fibrosis that produces results using the following formula: age (years) × AST (U/L)/(platelets (10^9^/L) × ALT (U/L)^1/2^) [17]. An FIB-4 index < 1.45 in the context of NAFLD excludes clinically significant hepatic fibrosis [18].

### Radiology assessments

All the subjects were examined using abdominal ultrasonography, and the radiologist was blinded to the study. Fatty liver was preliminarily diagnosed based on the signs of diffuse enhancement of the near echo, obvious attenuation of the far echo, contrast enhancement of the liver and kidney echo and unclear intrahepatic duct structure [12]. Magnetic resonance imaging-based proton density fat fraction (MRI-PDFF) with the IDEAL-IQ/Dixon sequence is considered a new acute and reproducible method to estimate the fat content of the whole liver and pancreas and the thickness of the abdominal subcutaneous tissue [19, 20], and some of the participants received MRI-PDFF estimations. We used a 3.0 T MRI with the following settings, as previously described: TE1 2.5 ms, TE2 3.7 ms, repetition time 5.47 ms, 5° flip angle, ± 504.0 kHz per pixel receiver bandwidth, and a slice thickness of 3.0 mm. The fat content was calculated in an irregularly shaped ROI covering the entire liver in 21 consecutive slices (max-area centered) of each patient, and patients were manually placed by two trained radiologists [21].

### Clinical follow-up and treatment

All patients received recommendations for lifestyle interventions according to the Dietary Reference Intakes [22], the Dietary Guidelines [23] and World Health Organization Global Strategy on Diet, Physical Activity and Health [24]. The patients were guided to adjust their food consumption and to exercise three times/week for 30 min per session by an easy-to-carry brochure with personalized exercise and dietary prescriptions based on sex, age, BMI, occupation and medical history. For patients with indications for drug therapy to treat hyperlipidemia, hypertension, hyperglycemia or hyperuricemia, pharmacological therapy was added as recommended by the guidelines [23–27]. Briefly, these treatments included metformin or insulin for glucose control, benzbromarone for uric acid control, renin–angiotensin blockers or a calcium channel blocker for blood pressure control, a statin for low-density lipoprotein cholesterol (LDL-C) control and fibrates for triglyceride control [25–28]. The prescription of specific agents was determined by the supervising physicians. Clinical follow-up and additional pertinent patient data are also provided. Some patients with a BMI ≥ 25 kg/m^2^ received orlistat (120 mg, three times daily) without additional treatment [21]. Orlistat intake was confirmed by the prescription and records of patient interviews during clinic visits.

The patients were subject to periodic reviews at 1, 3, 6, 9 and 12 months, and each visit was not postponed for one month after the prescribed time. At each follow-up visit, the anthropometric parameters, metabolic indexes and liver biochemical parameters of the patients were measured again. MRI-PDFF was only performed in 630 subjects every 6 months. Normalization of ALT and GGT was defined as values of these markers lower than the laboratory cut-off values, which was reported in the laboratory measurements section.

### Statistical analysis

All statistical calculations were conducted using SPSS statistics software (version 24.0, IBM, Chicago, IL, USA). The continuous variables are reported as means ± standard deviations (SD), and variables without a normal distribution are reported as medians with interquartile ranges (IQR). The Kruskal–Wallis rank sum test was used to compare non-normally distributed continuous variables between groups. Pearson’s chi-squared test was used to compare categorical data between groups. Multiple comparisons among groups were performed using ANOVA with the Bonferroni post hoc test. Logistic regression models with stepwise selection were used to estimate odds ratios (ORs) for the different stratifications of GGT levels in relation to metabolic parameters. A receiver operating characteristic (ROC) curve analysis was conducted to identify the factors predicting decreased GGT levels. P values for the trend (two-sided) were calculated and were considered statistically significant when they were less than 0.05.

## Results

### Baseline characteristics

**Fig. 1:**
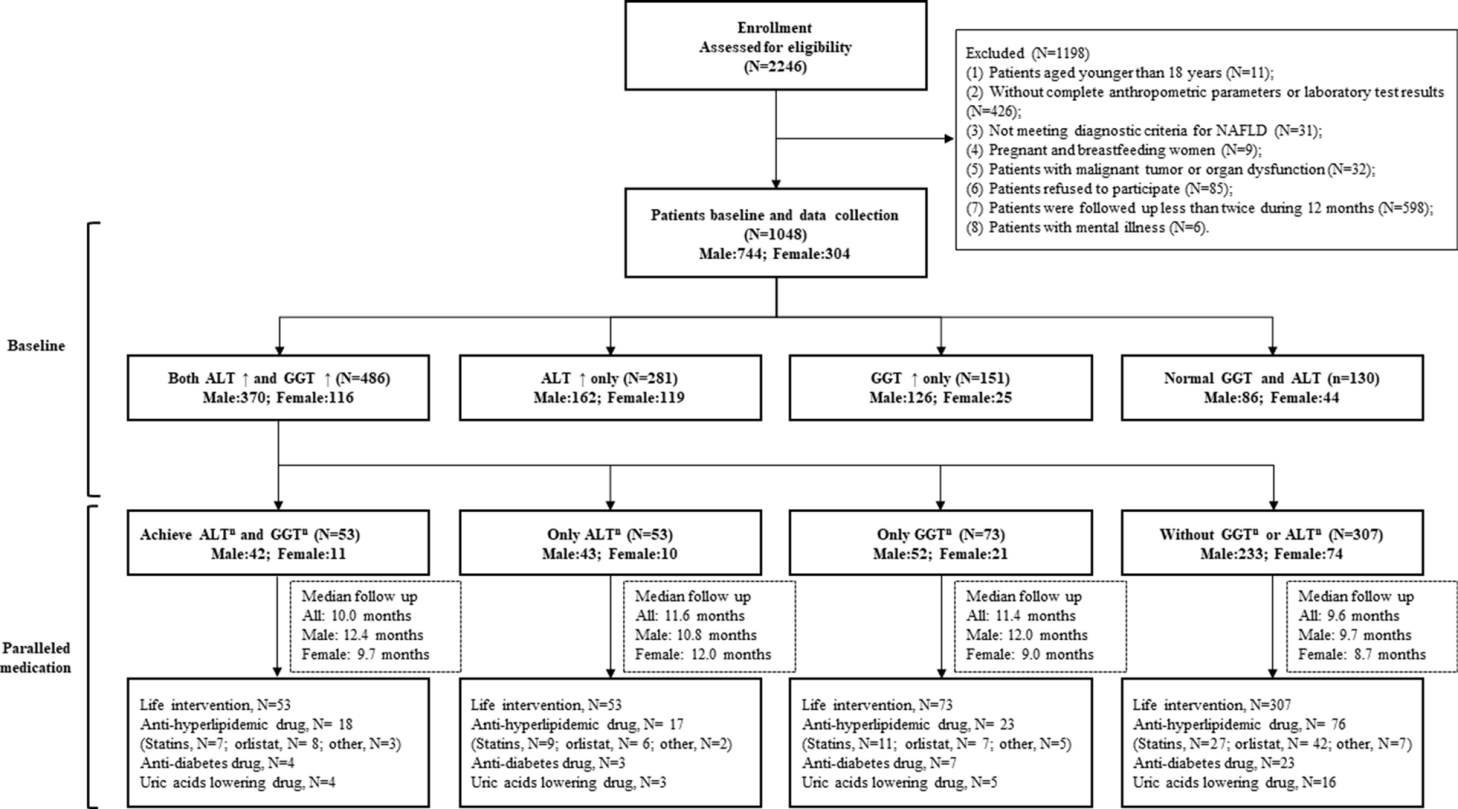
Flow diagram of participant recruitment, screening and allocation

The data of 2246 outpatients in the NAFLD clinic of the First Affiliated Hospital of Sun Yat-sen University from 2011 to 2019 were evaluated. 1198 cases were excluded, including 11 cases under 18 years old, 426 cases without complete anthropometric parameters or laboratory examination results, 31 cases not meeting the diagnostic criteria of NAFLD, 9 cases pregnant or breastfeeding, 32 cases with malignant tumor or organ dysfunction, 85 cases refusing to participate, 598 cases followed up less than twice within 12 months and 6 cases with mental illness (Fig. 1). A total of 1048 patients with NAFLD were included in this study and were divided into 4 groups based on the normalization of baseline ALT and/or GGT levels after follow-up: a both ALT and GGT abnormal group (n = 486), an ALT-only abnormal group (n = 281), a GGT-only abnormal group (n = 151), and a both ALT and GGT normal group (n = 130) (Fig. 1). No significant differences in fasting glucose level, prevalence of type 2 diabetes, hypertension, FIB-4 index, partial lipid metabolism or medications were observed between groups (Table 1, Additional file 1: Table S1). The group with abnormal levels in both ALT and GGT had higher BMIs (kg/m^2^) (median 26.4 vs. 26.4 vs. 25.3 vs. 25.3, *P* < 0.001, Table 1). Among the 1048 patients with NAFLD, 637 patients presented with abnormal GGT levels, and the percentage of patients with abnormal GGT levels was 60.7%. Meanwhile, 767 patients presented with abnormal ALT, and the percentage of patients with abnormal ALT levels was 73.2%. Compared with the other three groups, the group with abnormal levels of both ALT and GGT had higher liver function indexes, including GGT, ALT, AST, alkaline phosphatase, total bilirubin, direct bilirubin, total bile acid, lactate dehydrogenase, and glutamate dehydrogenase levels, except for choline esterase (all *P* < 0.05, Table 1). However, compared with the other three groups, significant increases in uric acid levels, fasting insulin levels, and HOMA-IR levels but not blood lipid metabolism were observed in the group with abnormal levels of both ALT and GGT (all *P* < 0.001, Table 1). Of the 630 patients with NAFLD who underwent MRI-PDFF, the group with abnormal levels of both ALT and GGT presented a significantly higher liver fat content (%) than the other three groups (median 15.2 vs. 15.5 vs. 8.3 vs. 9.4, *P* < 0.001, Table 1).

**Table 1:** Baseline characteristics of patients with NAFLD presenting different ALT and GGT statuses

Variables	Both GGT and ALT abnormal (N = 486)	ALT Abnormal only (N = 281)	GGT Abnormal only (N = 151)	Both GGT and ALT normal (N = 130)	P	Post-hoc					
						1^d^ versus 2^d^	1^d^ versus 3^d^	1^d^ versus 4^d^	2^d^ versus 3^d^	2^d^ versus 4^d^	3^d^ versus 4^d^
Age (years)	40.4 ± 12.5	44.1 ± 13.3	48.1 ± 11.2	48.2 ± 11.6	< 0.001	< 0.001	< 0.001	< 0.001	0.04	< 0.001	0.96
Male, n (%)	370 (76.1)	162 (57.7)	126 (83.4)	86 (66.1)	< 0.001	< 0.001	0.06	0.02	< 0.001	0.10	0.001
BMI (kg/m^2^)	26.4 (24.4,28.1)	26.4 (24.2,28.2)	25.3 (22.0,26.7)	25.3 (23.2,27.1)	< 0.001	0.83	< 0.001	< 0.001	0.001	< 0.001	0.45
Waist-hip ratio	0.90 (0.86,0.94)	0.89 (0.85,0.92)	0.91 (0.88,0.94)	0.89 (0.84,0.92)	0.01	0.05	0.15	0.01	0.02	0.31	0.01
Smoke, n (%)	94 (19.3)	29 (10.3)	43 (28.5)	16 (12.3)	< 0.001	0.001	0.02	0.06	< 0.001	0.55	0.001
*Complication, n (%)*											
Type 2 diabetes	37 (7.6)	22 (7.8)	5 (3.3)	13 (10.0)	0.16						
Hypertension	153 (31.5)	89 (31.7)	43 (28.5)	36 (27.7)	0.76						
*Liver biochemistry*											
GGT (U/L)	88 (65,144)	32 (26,42)	78 (59,129)	24 (18,32)	< 0.001	< 0.001	0.04	< 0.001	< 0.001	0.51	< 0.001
ALT (U/L)	80 (51,125)	41 (32,58)	22 (18,26)	19 (15,25)	< 0.001	< 0.001	< 0.001	< 0.001	0.03	< 0.001	0.89
AST (U/L)	45 (33,69)	31 (25,40)	22 (20,28)	20 (18,24)	< 0.001	< 0.001	< 0.001	< 0.001	0.38	0.01	0.61
Alkaline phosphatase (U/L)	83 (69,98)	73 (62,86)	81 (71,92)	70 (56,84)	< 0.001	0.001	0.04	< 0.001	0.56	0.36	0.28
Total bilirubin (umol/L)	13.4 (10.5,17.5)	12.2 (9.9,17.4)	10.7 (9.1,16.3)	12.9 (9.7,16.5)	0.04	0.03	0.11	0.02	0.60	0.81	0.70
Direct bilirubin (umol/L)	2.6 (1.9,3.5)	2.3 (1.8,3.4)	3.1 (1.7,3.4)	2.2 (1.7,3.0)	0.01	0.02	0.05	0.01	0.48	0.47	0.78
Total bile acid (umol/L)	2.9 (1.9,5.6)	2.1 (1.4,2.8)	2.8 (2.1,3.9)	2.3 (1.6,4.0)	0.01	0.01	0.50	0.01	0.51	0.88	0.46
Lactate dehydrogenase (U/L)	197 (174,224)	190 (171,218)	175 (162,199)	187 (165,203)	0.01	0.13	0.02	0.001	0.13	0.11	0.55
Choline esterase (U/L)	8936 (7878,9943)	8903 (8041,9912)	8197 (7596,9788)	9086 (8174,9887)	0.08						
Leucine arylamidase (U/L)	73 (65,91)	58 (51,65)	76 (65,90)	53 (49,60)	< 0.001	< 0.001	0.70	< 0.001	< 0.001	0.25	< 0.001
Glutamate dehydrogenase (U/L)	9.6 (5.3,13.9)	4.9 (2.8,7.8)	5.6 (4.2,7.4)	2.8 (2.1,4.0)	< 0.001	< 0.001	0.01	< 0.001	0.48	0.03	0.06
FIB-4 index	0.82 (0.53,1.16)	0.76 (0.55,1.12)	1.14 (0.82,1.38)	0.84 (0.61,1.15)	0.51						
*Metabolism*											
Uric acid (umol/L)	429 (364,493)	399 (325,463)	417 (360,482)	374 (314,440)	< 0.001	< 0.001	0.83	< 0.001	0.05	0.14	0.01
Hyperuricemia, n (%)	291 (59.9)	142 (50.5)	80 (52.9)	52 (40.0)	< 0.001	0.01	0.13	< 0.001	0.63	0.05	0.03
Cholesterol (mmol/L)	5.5 (4.9,6.2)	5.3 (4.7,5.9)	5.6 (4.7,6.4)	5.5 (4.8,6.3)	0.07						
Hyper-cholesterol, n (%)	211 (43.4)	87 (31.0)	70 (46.4)	54 (41.5)	0.01	0.001	0.53	0.70	0.01	0.04	0.42
Triglyceride (mmol/L)	2.0 (1.4,2.6)	1.7 (1.2,2.2)	2.0 (1.5,3.2)	1.7 (1.2,2.3)	< 0.001	< 0.001	0.03	0.01	< 0.001	0.42	0.001
Hyper-triglyceride, n (%)	294 (60.5)	134 (47.7)	95 (62.9)	95 (62.9)	< 0.001	0.001	0.59	0.01	0.01	0.99	0.01
HDL-cholesterol (mmol/L)	1.1 (1.0,1.4)	1.1 (1.0,1.3)	1.1 (1.0,1.3)	1.2 (1.0,1.4)	0.58						
LDL-cholesterol (mmol/L)	3.5 (2.9,4.0)	3.4 (2.9,4.0)	3.3 (2.7,3.9)	3.5 (2.8,4.1)	0.76						
Free fatty acid (mmol/L)	576 (426,731)	545 (437,702)	529 (414,619)	522 (427,595)	0.61						
Apolipoprotein-A (mmol/L)	1.2 (1.2,1.4)	1.2 (1.1,1.4)	1.3 (1.2,1.5)	1.3 (1.2,1.4)	0.50						
Apolipoprotein-B (mmol/L)	1.0 (0.9,1.2)	1.0 (0.8,1.1)	1.0 (0.8,1.2)	1.0 (0.8,1.1)	0.13						
Apolipoprotein-E (mmol/L)	48 (41,53)	45 (36,51)	47 (42,53)	44 (36,52)	0.18						
Lipoprotein-a (mmol/L)	165 (76,206)	189 (72,232)	177 (64,276)	176 (78,232)	0.01	0.03	0.04	0.01	0.36	0.65	0.53
Fasting glucose (mmol/L)	5.3 (4.8,5.7)	5.2 (4.8,5.5)	5.4 (4.8,6.0)	5.2 (4.8,5.6)	0.47						
Fasting insulin (uU/mL)	11.0 (8.5,12.8)	10.5 (8.0,12.1)	9.9 (7.2,11.0)	9.6 (6.0,11.0)	< 0.001	0.06	0.01	< 0.001	0.08	< 0.001	0.51
HOMA-IR	2.6 (1.9,3.1)	2.6 (1.8,2.8)	2.5 (1.9,2.6)	2.2 (1.4,2.6)	< 0.001	0.11	0.02	< 0.001	0.12	0.001	0.71
LFC^a^ (%)	15.2 (9.0,23.2)	15.5 (9.8,20.9)	8.3 (6.8,14.2)	9.4 (6.9,12.7)	< 0.001	0.55	0.001	< 0.001	0.01	< 0.001	0.81
PFC^b^ (%)	2.4 (1.6,3.6)	2.3 (1.6,3.2)	1.9 (1.7,3.2)	2.3 (1.5,3.2)	0.64						
ASFT^c^ (mm)	22 (17,28)	25 (20,31)	20 (17,26)	22 (18,29)	0.01	0.001	0.63	0.43	0.06	0.04	0.41
*Medication metabolism*											
Anti-hyperlipidemic drug, n (%)	146 (30.0)	85 (30.2)	48 (31.8)	39 (30.0)	0.98						
Anti-Diabetes drug, n (%)	31 (6.4)	17 (6.0)	5 (3.3)	8 (6.1)	0.56						
Uric acid lowering drug, n (%)	38 (7.8)	24 (8.5)	12 (8.0)	10 (7.7)	0.98						

Data are median (first quartile, third quartile), n (%), or mean ± SD (standard deviation)

Hyperuricemia was defined as uric acid level > 420 umol/L for male and > 360 umol/L for female; Hyper-cholesterol was defined as CHOL level > 5.7 mmol/L; Hyper-triglyceride was defined as TG level > 1.7 mmol/L

^a^Mean N = 610

^b^Mean N = 608

^c^Mean N = 610

^d^1—both GGT and ALT abnormal group; 2—ALT abnormal only group; 3—GGT abnormal only group; 4—both GGT and ALT normal group

For the 1048 NAFLD patients enrolled, there were 744 male and 304 female patients. Among male patients, 496 patients (68.3%) had abnormal GGT levels and 532 patients (73.3%) had abnormal ALT levels, while among female patients, 141 patients (43.8%) had abnormal GGT levels and 235 patients (73.0%) had abnormal ALT levels (Fig. 1). In both the male and female subgroups, the groups with abnormal levels of both ALT and GGT also presented higher BMIs and liver function indexes (all *P* < 0.05, Additional file 1: Table S2 and S3). In male patients, comparison with the other three groups indicated that the group with abnormal levels of both ALT and GGT exhibited significantly increased levels of cholesterol, triglyceride, apolipoprotein-A, apolipoprotein-B, and apolipoprotein-E; however, these lipid metabolites were not significantly increased in female patients (Additional file 1: Table S2 and S3). Furthermore, the levels of uric acid, fasting insulin, and HOMA-IR in male patients with abnormal levels of both ALT and GGT were higher than those in the other three groups, and only higher fasting insulin levels were observed in the female patients (Additional file 1: Table S2 and S3). In addition, there was no significant difference in the FIB-4 index between male and female patients (Additional file 1: Table S2 and S3).

### Comparison of metabolic control among the four groups stratified according to ALT and GGT levels

For the 1048 NAFLD patients followed up, 486 patients in the baseline group with abnormal levels of both ALT and GGT were further analyzed. Normalizations of both ALT and GGT were observed in 53 patients (10.9%), 53 patients (10.9%) exhibited ALT normalization alone, 73 patients (15.0%) exhibited GGT normalization alone, and 307 patients (63.2%) had persistently abnormal levels of both enzymes (Fig. 1). Compared with the other three groups, the weights and BMIs of group with normal levels of both ALT and GGT exhibited the greatest decrease (weight (%): 6.7 vs. 0.1 vs. 2.6 vs. 0.6, *P* < 0.001; BMI (kg/m^2^): 2.2 vs. 0.1 vs. 0.7 vs. 0.2, *P* < 0.001, Table 2). A similar trend was also observed in lipid metabolism-related parameters in the group with normalization of both ALT and GGT, including CHOL, LDL-C and APOE (all *P* < 0.05, Table 2). Significant differences in fasting insulin levels and HOMA-IR levels were observed among the four groups (all, *P* < 0.05, Table 2). When stratified by sex, the weight and BMI in the group with normalization of both ALT and GGT exhibited the greatest decrease compared with those in the other three groups in males (weight (%): 7.4 vs. 0.1 vs. 3.0 vs. 0.5, *P* < 0.001; BMI (kg/m^2^): 2.5 vs. 0.1 vs. 0.9 vs. 0.1, *P* < 0.001, Additional file 1: Table S4); however, this decrease was not observed in females. There were no significant differences in the parameters of lipid metabolism among the four groups in either sex-specific group except that APOE and FFA presented the largest extents of decrease in males and females with normalization of both ALT and GGT, respectively (Additional file 1: Table S4 and S5). Significant differences in fasting insulin and HOMA-IR levels were also observed between the four groups of males, but not females (all, *P* > 0.05, Additional file 1: Table S4 and S5). There were no significant differences in the FIB-4 index among the four groups in either sex-specific group (Additional file 1: Table S4 and S5).

**Table 2:** Biochemical and metabolic changes from baseline to month 12 in 486 patients with NAFLD presenting abnormal levels of both GGT and ALT at baseline

Variables	Both GGT and ALT normalization (N = 53)	ALT normalization only (N = 53)	GGT normalization only (N = 73)	Both GGT and ALT abnormal (N = 307)	*P*	Post-hoc					
						1^a^ versus 2^a^	1^a^ versus 3^a^	1^a^ versus 4^a^	2^a^ versus 3^a^	2^a^ versus 4^a^	3^a^ versus 4^a^
Weight change (kg	− 6.3 ± 12.1	− 0.1 ± 4.0	− 2.1 ± 4.0	− 0.5 ± 2.7	< 0.001	< 0.001	0.001	< 0.001	0.06	0.61	0.04
Weight change (%)	− 6.7 ± 9.5	0.1 ± 6.0	− 2.6 ± 4.9	− 0.6 ± 3.3	< 0.001	< 0.001	0.001	< 0.001	0.01	0.43	0.01
BMI (kg/m^2^)	− 2.2 ± 4.5	− 0.1 ± 0.8	− 0.7 ± 1.4	− 0.2 ± 1.0	< 0.001	< 0.001	0.001	< 0.001	0.10	0.88	0.03
Waist-hip ratio	− 0.03 ± 0.1	− 0.01 ± 0.1	− 0.01 ± 0.1	− 0.01 ± 0.1	0.01	0.02	0.01	0.001	0.92	0.70	0.75
ALT (U/L)	− 57 (− 74,− 32)	− 43 (− 73,− 23)	− 62 (− 96,− 25)	− 9 (− 41,0)	0.01	0.79	0.55	0.04	0.37	0.09	0.001
AST (U/L)	− 21 (− 30,− 7)	− 15 (− 34,− 3)	− 19 (− 50,− 5)	0 (− 17,2)	0.01	0.45	0.36	0.14	0.92	0.01	0.01
GGT (U/L)	− 46 (− 60,− 33)	− 27 (− 96,0)	− 33 (− 50,− 22)	− 5 (− 42,0)	0.01	0.12	0.63	0.28	0.22	0.01	0.05
ALP (U/L)	− 5 (− 12,0)	0 (− 13,5)	0 (− 16,6)	0 (− 7,2)	0.28						
TBil (umol/L)	− 0.8 ± 5.5	− 4.8 ± 13.6	0.3 ± 5.1	− 0.6 ± 7.6	0.11						
DBil (umol/L)	− 0.4 ± 2.7	− 1.6 ± 6.2	− 0.2 ± 1.9	− 0.3 ± 3.3	0.20						
TBA (umol/L)	− 0.1 ± 2.8	− 0.7 ± 3.5	− 0.2 ± 2.0	− 0.4 ± 6.7	0.97						
LDH (U/L)	− 10.4 ± 48.0	− 7.9 ± 25.1	− 12.1 ± 34.0	− 6.6 ± 28.9	0.69						
CHE (U/L)	− 344 (− 1431,− 179)	− 179 (− 334,0)	− 344 (− 806,− 179)	− 344 (− 334,0)	0.01	0.001	0.13	0.01	0.02	0.06	0.32
LAP (U/L)	− 2.1 ± 20.4	− 7.2 ± 15.4	− 3.1 ± 12.1	− 4.5 ± 16.6	0.57						
GLDH (U/L)	− 0.8 ± 6.9	− 1.3 ± 5.7	− 1.3 ± 3.9	− 1.1 ± 7.1	0.98						
FIB-4 index	0.1 ± 1.1	− 0.1 ± 0.3	− 0.1 ± 0.4	0.1 ± 0.6	0.80						
UA (umol/L)	− 29 (− 100,0)	− 3 (− 92,15)	− 19 (− 61,0)	0 (− 24,0)	0.13						
CHOL (mmol/L)	− 1.0 ± 1.7	− 0.2 ± 1.1	− 0.7 ± 1.5	− 0.3 ± 1.2	0.02	0.02	0.27	0.01	0.14	0.70	0.10
TG (mmol/L)	− 0.6 ± 1.2	− 0.3 ± 0.7	− 0.4 ± 0.9	− 0.2 ± 0.9	0.33						
HDL-C (mmol/L)	− 0.1 ± 0.3	− 0.1 ± 0.2	− 0.1 ± 0.4	− 0.1 ± 0.5	0.84						
LDL-C (mmol/L)	− 0.7 ± 1.2	− 0.2 ± 0.7	− 0.3 ± 0.8	− 0.1 ± 0.9	0.01	0.04	0.04	0.001	0.84	0.54	0.31
FFA (mmol/L)	− 56 (− 226,0)	− 0 (− 56,0)	0 (− 56,0)	− 53 (− 56,0)	0.05						
APOA (mmol/L)	− 0.1 ± 0.3	0.1 ± 0.3	− 0.1 ± 0.2	− 0.1 ± 0.2	0.46						
APOB (mmol/L)	− 0.1 ± 0.3	− 0.1 ± 0.2	− 0.1 ± 0.2	− 0.2 ± 0.2	0.66						
APOE (mmol/L)	− 8 (− 14,0)	− 1 (− 7,0)	− 1 (− 9,0)	0 (− 7,0)	0.01	0.06	0.01	0.01	0.38	0.49	0.67
LPA (mmol/L)	7 (0,42)	6 (0,27)	0 (− 11,16)	0 (0,11)	0.62						
FBG (mmol/L)	− 0.4 ± 1.5	− 0.2 ± 1.0	− 0.3 ± 0.6	− 0.2 ± 0.6	0.23						
FINS (uU/mL)	− 2.0 ± 4.5	− 1.2 ± 3.6	− 0.2 ± 3.4	0.1 ± 4.2	0.04	0.41	0.05	0.01	0.27	0.11	0.74
HOMA-IR	− 0.7 ± 1.2	− 0.5 ± 1.0	− 0.2 ± 0.9	− 0.1 ± 1.1	0.01	0.57	0.04	0.01	0.15	0.03	0.63

Data are median (first quartile, third quartile) or mean ± SD (standard deviation)

^a^1—both GGT and ALT normalization group; 2—ALT normalization only group; 3—GGT normalization only group; 4—both GGT and ALT abnormal group

**Fig. 2:**
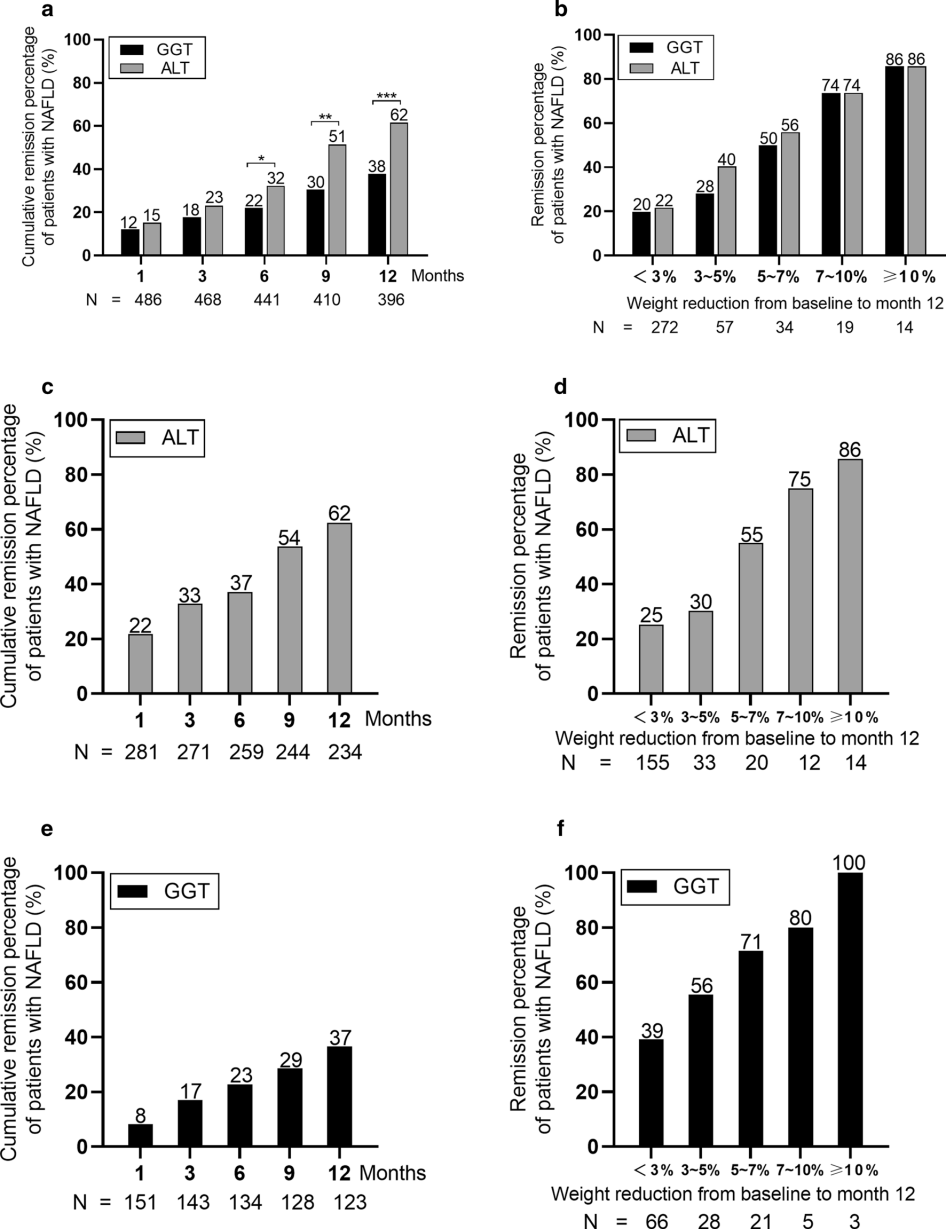
The cumulative normalization rates of ALT and GGT levels after 12 months of treatment. **a** The cumulative normalization rates of ALT and GGT levels at baseline in the group with abnormal levels of both ALT and GGT. **b** The relationships between the weight change ratio and ALT or GGT normalization rates at baseline in the group with abnormal levels of both ALT and GGT. **c** The cumulative normalization rate of ALT levels in the baseline ALT-only abnormal group. **d** The relationship between the weight change ratio and the ALT normalization rate in the baseline ALT-only abnormal group. **e** The cumulative normalization rate of GGT levels in the baseline GGT-only abnormal group. **f** The relationship between the weight change ratio and the GGT normalization rate in the baseline GGT-only abnormal group. *P < 0.05, **P < 0.01, ***P < 0.001

Patients in the baseline group with abnormal levels of both ALT and GGT were monitored for 12 months to detect the normalization rates of GGT and ALT levels during treatment. After 6, 9, and 12 months of follow-up, the cumulative normalization rates of GGT levels were 22%, 30% and 38%, and the cumulative normalization rates of ALT levels were 32%, 51%, and 62% (all *P* < 0.05, Fig. 2a), respectively. Similar trends of the cumulative normalization rates for GGT and ALT levels at the same time points also existed (20%, 29% and 31% of GGT and 27%, 41% and 53% of ALT in males, all *P* < 0.05, Additional file 2a). After 12 months of follow-up, there was no significant difference in the cumulative normalization rates of GGT and ALT levels in female patients (Additional file 2b).

These patients were further divided into 5 groups according to weight change after 12 months of treatment as follows: < 3% (n = 272), 3–5% (n = 57), 5–7% (n = 34), 7–10% (n = 19) and ≥ 10% (n = 14) (Fig. 2b). In patients with a weight change ratio < 3%, the cumulative normalization rates of GGT levels were significantly lower than those in the other four groups (Fig. 2b, Additional file 2c and d).

We also described the changes in normalization rates in the ALT-only abnormal group and the GGT-only abnormal group after 12 months of treatment. For the patients in the ALT-only abnormal group, the cumulative normalization rate of ALT levels was 62% after 12 months of follow-up (Fig. 2c), with rates of 65% and 58% in male and female patients, respectively (Additional file 2e and f). The lowest cumulative normalization rates were observed in patients with weight change ratios < 3% (Fig. 2d, Additional file 2g and h). In the GGT-only abnormal group, the cumulative normalization rate of GGT levels after 12 months of follow-up was 37%, with rates of 34% and 43% in male and female patients (Fig. 2e, Additional file 2i and j); similar associations with weight change were found in the overall and both sex-specific cohorts (Fig. 2f, Additional file 2k and l).

### Predictors of GGT remission in patients with NAFLD after 12 months of treatment

**Fig. 3:**
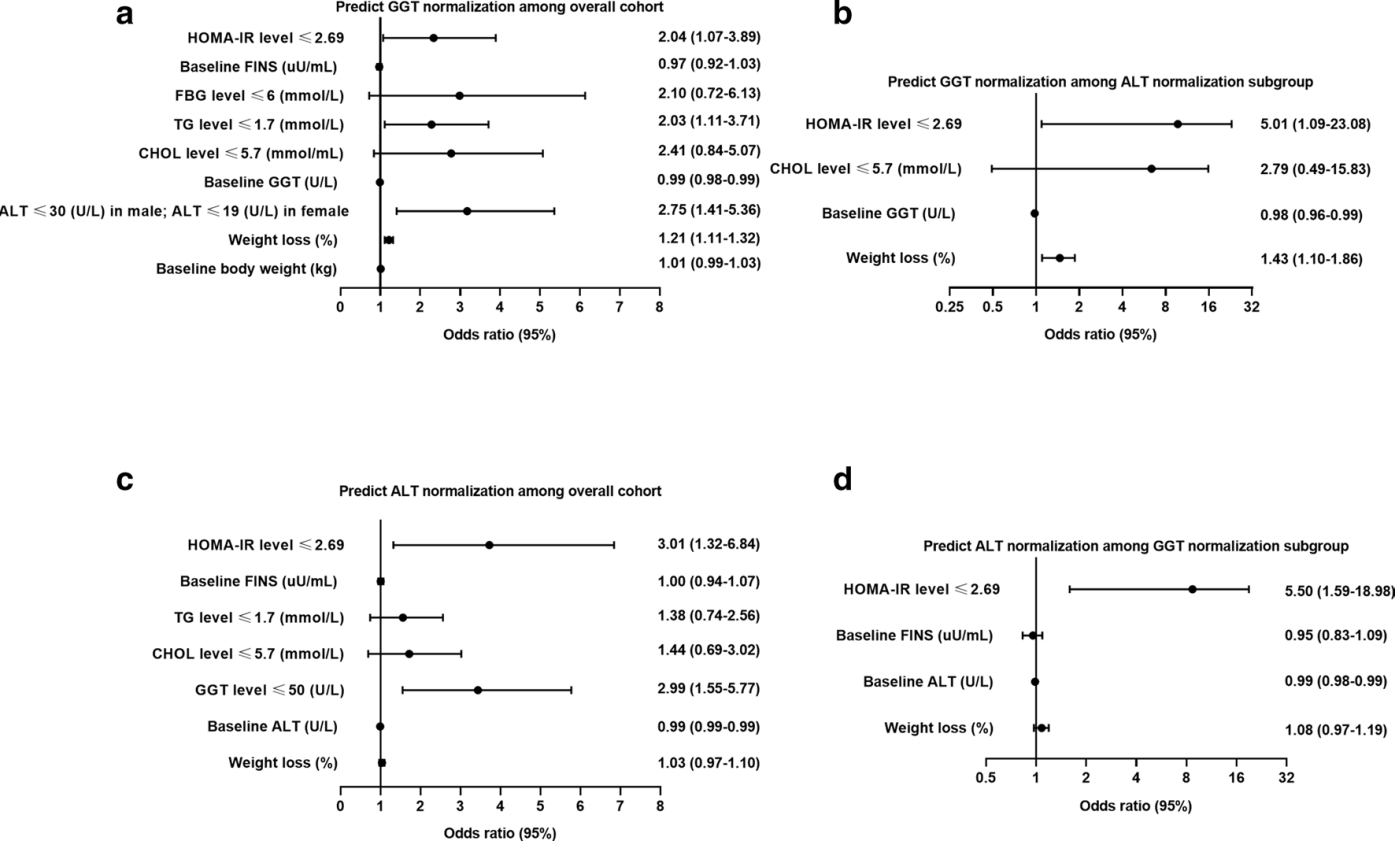
Forest plot predicting the normalization of GGT and ALT. Prediction of GGT normalization in all populations (**a**) and in the ALT normalization population (**b**). Prediction of ALT normalization in all populations (**c**) and in the GGT normalization population (**d**)

A univariate logistic regression analysis showed that weight loss, baseline body weight, smoking status, drinking status, baseline GGT levels and the normalization of ALT, CHOL, TG and FBG levels after treatment were independent factors influencing the recovery of GGT levels in 486 patients with both baseline ALT and GGT abnormalities (Fig. 3a, Additional file 1: Table S6). After multivariate adjustment, weight loss (OR = 1.21, 95% CI 1.11–1.32, *P* < 0.001), ALT normalization (OR = 2.75, 95% CI 1.41–5.36, *P* = 0.01), and decreases in TG and HOMA-IR to normal levels (OR = 2.03, 95% CI 1.11–3.71, *P* = 0.02; OR = 2.04, 95% CI 1.07–3.89, *P* = 0.03) were independent protective factors for GGT normalization, while elevated baseline GGT (OR = 0.99, 95% CI 0.98–0.99, *P* = 0.01) was identified as a risk factor (Fig. 3a, Additional file 1: Table S6). In the subgroup of 106 patients with NAFLD whose ALT levels returned to normal after 12 months of treatment, the multivariate models showed that weight loss (OR = 1.43, 95% CI: 1.10–1.86, *P* = 0.01) and a decrease in HOMA-IR to a normal level (OR = 5.01, 95% CI 1.09–23.08, *P* = 0.04) remained independent factors associated with GGT normalization (Fig. 3b, Additional file 1: Table S6).

When univariate logistic regression analysis was applied to the male subgroup, baseline body weight, weight loss, baseline GGT levels, and the normalization of ALT, CHOL, TG, FBG, and HOMA-IR after treatment were independent factors influencing the recovery of GGT levels (Additional file 3a, Additional file 1: Table S7). After multivariate adjustment, only weight loss (OR = 1.22, 95% CI 1.09–1.37, *P* < 0.001), normalization of ALT level (OR = 3.43, 95% CI 1.66–7.07, *P* = 0.01), and decreases in TG and HOMA-IR to normal levels (OR = 2.31, 95% CI 1.19–4.50, *P* = 0.02; OR = 2.65, 95% CI 1.34–5.24, *P* = 0.01) remained significant for GGT normalization (Additional file 3a, Additional file 1: Table S7). Baseline GGT level (OR = 0.99, 95% CI 0.98–0.99, *P* = 0.01) was found to be an independent risk factor associated with GGT normalization. In the subgroup of 85 male patients with NAFLD whose ALT levels returned to normal after 12 months of treatment, multivariate regression analysis identified that weight loss (OR = 1.51, 95% CI 1.11–2.06, *P* = 0.01) remained an independent factor associated with GGT normalization (Additional file 3b, Additional file 1: Table S7). For 116 female patients with abnormal baseline ALT and GGT levels, multivariate regression analysis showed that the normalization of CHOL levels after treatment was an independent factor affecting the recovery of GGT levels (Additional file 3e, Additional file 1: Table S8).

**Fig. 4:**
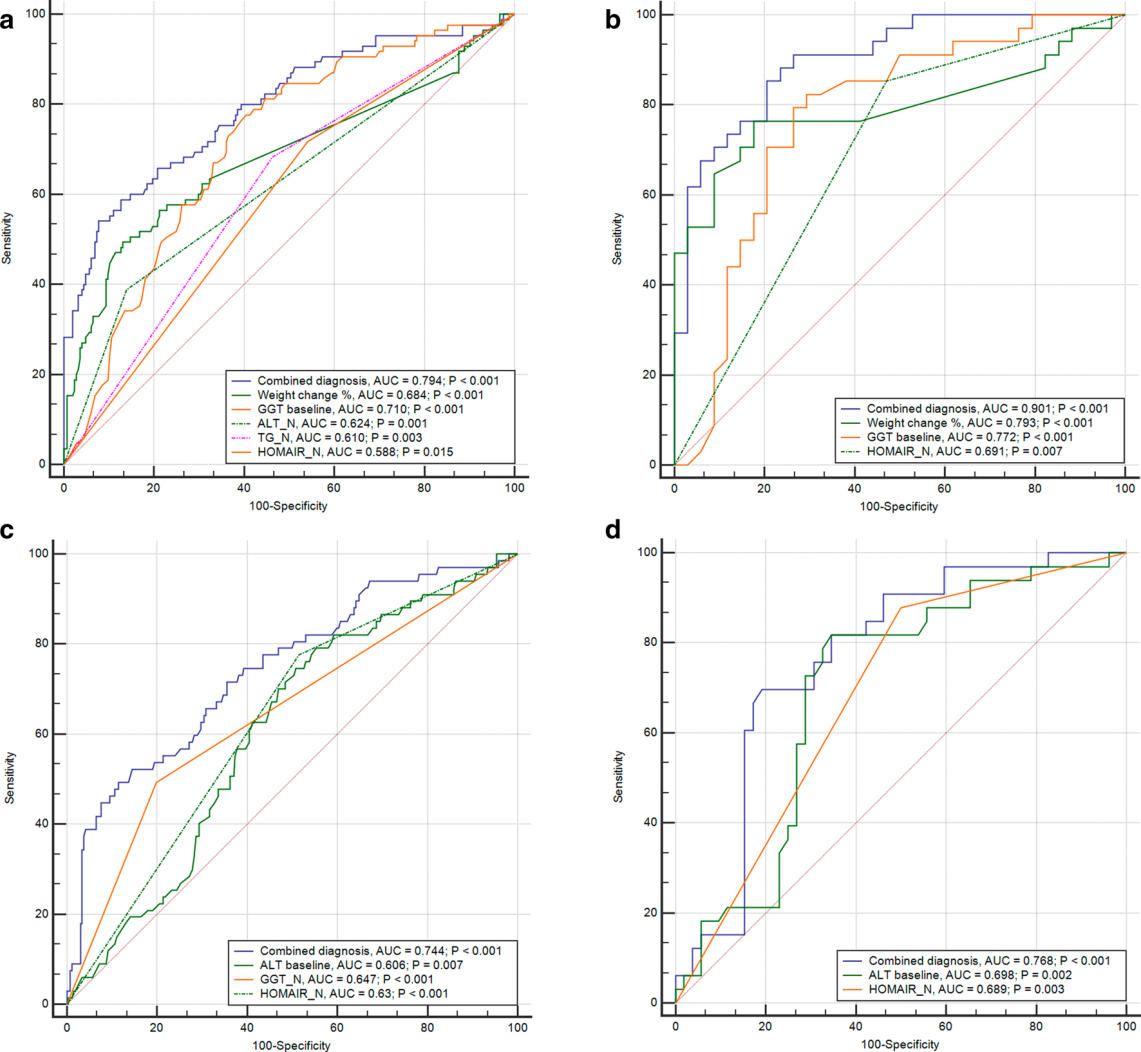
ROC curves predicting the normalization of GGT and ALT. Prediction of GGT normalization in all populations (**a**) and in the normal ALT population (**b**). Prediction of ALT normalization in all populations (**c**) and in the normal GGT population (**d**). ALT_N: ALT decreased to normal was defined as ALT level ≤ 30 U/L for male and ≤ 19 U/L for female; TG_N: TG decreased to normal was defined as TG level ≤ 1.7 mmol/L; GGT_N: GGT decreased to normal was defined as GGT level ≤ 50 U/L; HOMA-IR_N: HOMA-IR decreased to normal was defined as HOMA-IR ≤ 2.69

We further explored the application of the factors identified by the logistic model as predictors of GGT normalization and found that weight change, baseline GGT level and normalizations of ALT, TG and HOMA-IR exhibited significant values for areas under the ROC curves (*P* < 0.01, Fig. 4a). The combination of these factors obtained an AUC of 0.794 (*P* < 0.001, Fig. 4a). For patients with ALT remission, we identified that a weight change, baseline GGT levels, normalization of HOMA-IR levels and their combination were able to predict GGT remission with AUCs of 0.793, 0.772, 0.691 and 0.901, respectively (all, *P* < 0.01, Fig. 4b).

For the predictors of GGT normalization in male patients, the results indicated that the baseline GGT level, weight change, ALT normalization, TG and HOMA-IR decreased to normal levels, corresponding to significant values for areas under the ROC curves (*P* < 0.01, Additional file 4a). The combination of these factors corresponded to an area under the curve of 0.830. Baseline GGT level, weight change and their combination were able to predict GGT remission in male patients with ALT remission with AUCs of 0.827, 0.818, and 0.897, respectively (all *P* < 0.001, Additional file 4b). The normalization of CHOL levels after treatment predicted GGT normalization in female patients with an AUC of 0.623 (*P* = 0.004, Additional file 4e).

### Predictors of ALT remission in patients with NAFLD after 12 months of treatment

The univariate logistic regression analysis revealed that changes in weight loss, baseline levels of ALT and FINS and the normalization of GGT, CHOL, TG and HOMA-IR after treatment were independent factors influencing the recovery of ALT levels in subjects with abnormal levels of both ALT and GGT at baseline (Fig. 3c, Additional file 1: Table S9). After the multivariate analysis, GGT and HOMA-IR levels decreased to normal (OR = 2.99, 95% CI 1.55–5.77, *P* = 0.001; OR = 3.01, 95% CI 1.32–6.84, *P* = 0.01) remained independent (Fig. 3c, Additional file 1: Table S9). In 126 patients with NAFLD whose GGT levels returned to normal after 12 months of treatment, only HOMA-IR normalization was a statistically significant factor influencing the recovery of ALT levels (OR = 5.50, 95% CI 1.59–18.98, *P* = 0.01) (Fig. 3d, Additional file 1: Table S9).

Univariate logistic regression analysis was also conducted on 370 male patients with abnormal levels of both ALT and GGT at baseline, and it showed that the changes in weight loss, total bilirubin level, baseline FINS level, and the normalization of GGT, CHOL, TG, and HOMA-IR after treatment were independent factors influencing the recovery of ALT levels (Additional file 3c, Additional file 1: Table S10). Normalizations of GGT and HOMA-IR levels after treatment were independent protective factors for ALT normalization. In 94 male patients with NAFLD whose GGT levels returned to normal after 12 months of treatment, baseline ALT level was an independent risk factor associated with ALT normalization. Baseline LDL-C level and normalization of the HOMA-IR level after treatment were independent protective factors influencing the recovery of ALT levels (Additional file 3d, Additional file 1: Table S10). For 116 female patients with abnormal baseline ALT and GGT levels, multivariate regression analysis revealed that no metabolic factors affected the recovery of ALT levels (Additional file 1: Table S11).

The accuracy of these factors derived from the logistic model was also estimated to predict ALT normalization. Baseline ALT level and normalizations of GGT and HOMA-IR exhibited significant values for areas under the ROC curves (*P* < 0.01, Fig. 4c). The combination of these three factors achieved a higher AUC of 0.744 (*P* < 0.001, Fig. 4c). The combination of ALT baseline and HOMA-IR normalization had a significant AUC of 0.768 in the subgroup of patients who achieved GGT normalization (*P* < 0.001, Fig. 4d).

We also compared the accuracy of the factors derived from the logistic model for the prediction of ALT normalization in male patients. Normalizations of GGT and HOMA-IR levels after treatment corresponded to significant values for areas under the ROC curves (*P* < 0.05, Additional file 4c). The combination of these factors corresponded to an AUC of 0.688. Baseline ALT and LDL-C levels and normalization of the HOMA-IR level after treatment were able to predict ALT remission in male patients with GGT normalization with AUCs of 0.714, 0.650, and 0.697, respectively (all, *P* < 0.05, Additional file 4d). The combination of these factors corresponded to an AUC of 0.856.

### Associations of the changes in GGT levels and liver fat content after 12 months of treatment

**Fig. 5:**
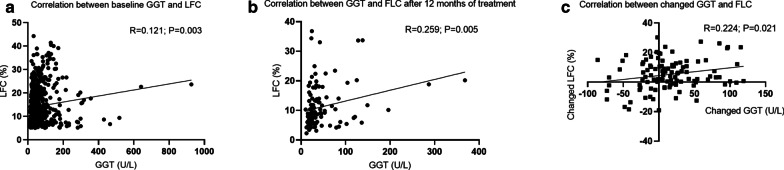
Correlations between liver fat content determined using MRI-PDFF and GGT levels in 630 patients with NAFLD. Scatter plots of the correlations between GGT levels and the liver fat content **a** at baseline and **b** after 12 months of treatment and **c** the changes from baseline to month 12

Patients in this study were recruited from 1 January 2011 to 31 December 2019, whereas MRI-PDFF was available in our NAFLD center until January 2015. Among 1048 NAFLD patients diagnosed by ultrasound, 630 patients (60%) underwent MRI-PDFF examination at baseline, and 221 patients received repeated MRI-PDFF measurements in the sixth month. We identified a weak correlation between the changes in GGT levels and the changes in the LFC (R = 0.224, *P* = 0.021, Fig. 5c). However, this significant correlation disappeared in male or female patients (R = 0.215, *P* = 0.057 for males and R = 0.255, *P* = 0.278 for females, Additional file 5e and f).

### Comparison of metabolic control between groups stratified by FIB-4 index and ALT and GGT levels

A Fib4 index lower than the recommended lower limit (< 1.45) was considered to exclude liver fibrosis. In the patients with a baseline FIB-4 index < 1.45, after 6, 9, and 12 months of follow-up, the cumulative normalization rates of GGT levels were 22%, 31% and 34%, and the cumulative normalization rates of ALT levels were 29%, 44%, and 55% (all *P* < 0.05, Additional file 6a), respectively. Similar trends of the cumulative normalization rates of GGT and ALT levels at the same time points also existed (20%, 29% and 31% of GGT and 28%, 43%, and 55% of ALT in males, all *P* < 0.05, Additional file 6b). After 12 months of follow-up, there were no significant differences in the cumulative normalization rates of GGT and ALT levels in female patients (Additional file 6c). In patients with a baseline FIB-4 index ≥ 1.45, there were no significant differences in the cumulative normalization rates of GGT and ALT levels between male and female patients after 12 months of follow-up (Additional file 6j, k and l).

We also described the changes in normalization rates in the ALT-only and GGT-only abnormal groups after 12 months of treatment. For the patients with a baseline FIB-4 index < 1.45 in the ALT-only abnormal group, the cumulative normalization rate of ALT levels was 63% after 12 months of follow-up (Additional file 6d), with rates of 66% and 58% in male and female patients, respectively (Additional file 6e and f). In the GGT-only abnormal group, the cumulative normalization rate of GGT levels after 12 months of follow-up was 37%, with rates of 36% and 42% in male and female patients, respectively (Additional file 6g, h and i). In patients with a baseline FIB-4 index ≥ 1.45, the cumulative normalization rates of ALT and GGT levels were 56% and 38%, respectively, after 12 months of follow-up (Additional file 6m and p). We identified a significant correlation between the baseline GGT levels and baseline FIB-4 index (R = 0.312, *P* < 0.001, Additional file 7a). However, this significant correlation disappeared in changed GGT levels and changed FIB-4 index (R = 0.126, *P* = 0.050, Additional file 7g).

## Discussion

In this retrospective cohort study of 1048 consecutive patients with NAFLD with and without baseline increases in liver enzyme levels, we observed an association between increased GGT levels at baseline and higher triglyceride and cholesterol levels compared to patients with abnormal ALT levels alone. Moreover, for patients with concurrently increased ALT and GGT levels during follow-up, a lower normalization rate was observed for GGT than for ALT. Significant associations between the normalization of GGT levels and target ALT, triglyceride and HOMA-IR levels, but not LDL-C or HDL-C levels, were observed in addition to weight loss. As shown in the present study, metabolic control was an independent protective factor for GGT level normalization in NAFLD management.

GGT and ALT levels have been well accepted as important variables for screening NAFLD because both indicators are tested using a simple routine method in clinical or epidemiologic settings [7, 29, 30]. In the present study, the prevalence of simultaneous GGT and ALT abnormalities in Chinese patients with NAFLD was 46.4%, which was similar to another large-scale cross-sectional study conducted in China, in which over 50% of patients with NAFLD presented abnormal serum liver enzyme levels [31]. Although ALT, AST and GGT are considered markers of liver injury because they are released from disrupted hepatocytes in patients with NAFLD, the levels of GGT, a key enzyme involved in glutathione and cysteine metabolism, are increased in not only patients with NAFLD but also patients with many other conditions, including oxidative stress, cholestatic liver disease and ethanol exposure [32]. Increased bile pressure secondary to steatosis has been identified as an important pathogenic mechanism in patients with NAFLD and oxidative stress and may explain the higher specificity of this parameter for NAFLD than that of ALT [33].

Normalizations of both GGT and ALT were proposed as predictors of histological improvement, especially inflammation, in the routine management of patients with NAFLD [11]. For predicting fibrosis, liver enzyme levels have less certainty. A previous study reviewed 515 patients with biopsy-proven NAFLD and showed that individuals with normal or abnormal transaminase levels had a similar prevalence of advanced fibrosis [34]. Another retrospective cohort study derived from the Corporate Data Warehouse of the Veterans Health Administration covering 28,208 samples with at least a 7‐year follow‐up demonstrated that the risk of progression to cirrhosis in the steatosis group with normal ALT levels was much lower than that in the group with both steatosis and abnormal ALT levels but was not different from the risk among those without steatosis or elevated ALT [35]. The dynamic association between liver biochemical markers and disease progression is complex and multifactorial [11, 36]. However, the differences in dynamic changes between serum GGT and ALT activity levels have seldom been reported during NAFLD treatment [37]. In this study, we described distinct decreasing trends in ALT and GGT levels after the intervention was initiated, with GGT levels presenting a much slower restoration than ALT levels. Interestingly, in addition to the common factor of weight loss, different factors have also been shown to be independently associated with ALT or GGT normalization. Persistent GGT abnormalities tended to be associated with poor metabolic control, including triglyceride levels and HOMA-IR levels.

Previous studies have indicated that GGT is a sensitive marker of insulin resistance in adults. A cross-sectional study in Italians showed that insulin resistance determined as HOMA-IR in obesity is a risk factor for increased levels of liver enzymes in NAFLD, with a significant correlation observed [38], while another study further suggested that serum GGT levels were an independent predictor of HOMA-IR in addition to steatosis [39]. Moreover, the results of a study in healthy individuals demonstrated that GGT levels were associated with higher insulin secretion rates measured by euglycemic-hyper-insulinemic clamp, reduced endogenous clearance of insulin and hepatic insulin extraction during the OGTT, and glucagon concentration [40]. Data from a longitudinal epidemiological study with 3545 participants showed that high levels of GGT were associated with the 3-year incidence of metabolic syndrome components, and this association was attenuated by the severity of insulin resistance [41]. Emerging evidence also supports a close association between metabolic dysregulation, steatosis degree and GGT levels in patients with NAFLD [42]. Another Brazilian study reported an increase in GGT levels as the degree of steatosis increased, as insulin resistance has been identified as the acknowledged mechanism driving hepatic de novo lipid synthesis, which would result in the increased release of fatty acids and derived products, such as triglycerides and cholesterol [42]. Using a multivariate linear regression analysis, a significant positive correlation was observed between GGT and HOMA-IR (standard β = 0.252) in a population-based cross-sectional study conducted in a Chinese population [42]. In the Framingham offspring study with a 20-year follow-up period, GGT level quartiles at baseline exhibited dose–response effects on the occurrence of cardiac risk factors, including serum lipid profiles, blood glucose levels and the development of diabetes [43]. Our research expanded on a previous cross-sectional study investigating the correlation between changes in GGT levels and IR and related metabolic dysfunction for the first time, and our study provided novel findings that the degree of IR decrease to normal and other related indexes that were reduced to target levels by treatment are potential predictors of restored GGT levels, suggesting that the clinical value of GGT differs from ALT as a noninvasive monitoring parameter to directly estimate the posttreatment severity of NAFLD [11].

A robust line of current evidence has identified NAFLD as a sexually dimorphic disease. Sex differences occur in the prevalence, risk factors, disease progression, and metabolic comorbidities of NAFLD, and these sex disparities manifest themselves in terms of not only hormone levels or menopause status but also fat distribution and even sociocultural status [44]. During lifestyle modification therapy, males reported that a relatively modest weight loss (7–10%) was needed when gaining the same extent of beneficial histological improvements, while a larger extent of weight loss (> 10%) was required in women [45]. The cut-off values of ALT suggest that different normal ranges should be applied by sex, such as 30 U/L for men and 19 U/L for women. Therefore, our results pertaining to NAFLD and the markers of ALT and GGT as treatment responses must be rerun separately for men and women. The current results support that the distinct GGT and ALT normalization patterns share the same trends in the overall samples and both sex subgroups, and the proportions of patients achieving GGT normalization were close in both sex subgroups. These findings indicated that better metabolic abnormality control provided similar effects of treatment responses in NAFLD despite the sex differences.

It is well known that statins and antidiabetic medication drugs play crucial roles in the management of NAF with dyslipidemia and diabetes [46, 47]. Regarding statins, although their administration has not been acknowledged as a specific therapy for NAFLD, post hoc analyses of large prospective randomized controlled trials suggest that statins may confer a substantial improvement in abnormal aminotransferase levels without an additional risk of hepatotoxicity [46]. Several types of antidiabetic drugs, including metformin, glucagon-like peptide-1 receptor agonists and dipeptidyl peptidase-4 inhibitors, were also associated with the normalization of enzyme levels in NAFLD by inhibiting liver inflammation and improving insulin resistance [46, 47]. The distribution of types of antidiabetic drugs and statins prescribed to our subjects did not present significant differences in the subgroups achieving the normalization of ALT or GGT alone or concurrently. The current results further support that the treatment of targets of metabolic parameters would be beneficial to the treatment response.

The current study had several limitations. First, the relationships between liver biochemical parameters and histological inflammation or the degree of fibrosis in patients with NAFLD were not estimated because the majority of these patients did not undergo liver biopsy in the follow-up period. Previous studies have clearly indicated that overreliance on liver enzymes is a mistake, as the diagnosis of NAFLD with advanced fibrosis or inflammation is not ruled out by the presence of normal liver enzymes, and the percentage of patients with normal ALT or GGT levels was 53.6% at baseline. Second, the findings of our study may not be generalizable to patients with NAFLD and other known causes of GGT abnormalities. A subset of patients may have their GGT values reduced owing to a reduced intake of alcohol, which was probably hidden at baseline. Third, MRI-PDFF is normally a very sensitive method for the diagnosis of hepatic steatosis. However, some of the patients were diagnosed by ultrasonography, which may cause patients with lower hepatic steatosis to be missed. Fourth, simple hepatic steatosis is usually benign and is not usually associated with increased liver enzymes. The main prognostic factor is the presence of hepatic fibrosis. For the estimation of hepatic fibrosis, FIB-4 index and NFS are reliable scoring systems also recommended in major guidelines [1, 48, 49]. and they have been shown to perform accurately in patients both with normal and abnormal transaminase levels [50]. However, their diagnostic ability lies on accurate exclusion of advanced fibrosis rather than detection of it [51]. Therefore, the association of advanced fibrosis and GGT normalization during NAFLD monitoring remain histology confirmation. Finally, additional studies are warranted to clarify the relationships between the levels of GGT and other liver enzymes when monitoring NAFLD severity.

## Conclusions

Overall, although serum levels of both ALT and GGT are useful markers for the noninvasive surveillance of the hepatic histological response to treatment in patients with NAFLD, we first compared the clinical significance among different liver biochemistry markers, including serum ALT and GGT, in the management of metabolic abnormalities during NAFLD monitoring and demonstrated that their associations with metabolic parameters were different. Our study emphasized that the rectification of dyslipidemia and insulin resistance levels may be necessary to achieve GGT normalization, which may reduce inflammation and prevent the deterioration of fibrosis in patients with NAFLD [11, 12]. In contrast, NAFLD patients who show a return to normal GGT levels may benefit from earlier intensive interventions with drugs for metabolic control in combination with an appropriate diet and exercise strategy.

## Supplementary Information

**Additional file 1.** Summary of all additional tables (S1–S11). (**Table S1**) Medication status and follow-up time of NAFLD patients; Baseline characteristics of male (**Table S2**) and female (**Table S3**) NAFLD patients presenting different ALT and GGT statuses; Biochemical and metabolic changes from baseline to month 12 in male (**Table S4**) and female (**Table S5**) NAFLD patients presenting abnormal levels of both GGT and ALT at baseline; Factors associated with GGT normalization in overall patients (**Table S6**), male patients (**Table S7**) and female patients (**Table S8**) with NAFLD after 12 months of treatment predicted by the logistic regression model; Factors associated with ALT normalization in overall patients (**Table S9**), male patients (**Table S10**) and female patients (**Table S11**) with NAFLD after 12 months of treatment predicted by the logistic regression model.

**Additional file 2.** Cumulative normalization rates of ALT and GGT levels after 12 months of treatment. Cumulative normalization rates of ALT or GGT levels in the groups of **(a)** male patients and **(b)** female patients with abnormal levels of both ALT and GGT; **(e)** the male group and **(f)** the female group with abnormal ALT levels only; and **(i)** the male group and **(j)** the female group with abnormal GGT levels only. Relationships between the weight change ratio and ALT or GGT normalization rates **(c)** in the male group and **(d)** the female group with abnormal levels of both ALT and GGT; **(g)** the male group and **(h)** the female group with abnormal ALT levels only; and **(k)** the male group and **(l)** the female group with abnormal GGT levels only.

**Additional file 3.** Forest plot predicting the normalization of GGT and ALT. Prediction of GGT normalization in all male populations **(a)** and the normal ALT male population **(b)**; prediction of ALT normalization in all male populations **(c)** and the normal GGT male population **(d)**; prediction of GGT normalization in all female populations **(e)**.

**Additional file 4.** ROC curves predicting the normalization of GGT and ALT. Prediction of GGT normalization in all male populations **(a)** and the normal ALT male population **(b)**; prediction of ALT normalization in all male populations **(c)** and the normal GGT male population **(d)**; prediction of GGT normalization in all female populations **(e)**.

**Additional file 5.** Correlations between liver fat content determined using MRI-PDFF and GGT levels in 630 patients (461 male and 169 female) with NAFLD. Scatter plots of the correlations between GGT levels and the liver fat content in male patients **(a)** at baseline and **(c)** after 12 months of treatment and **(e)** the changes from baseline to month 12; and in female patients **(b)** at baseline and **(d)** after 12 months of treatment and **(f)** the changes from baseline to month 12.

**Additional file 6.** Cumulative normalization rates of ALT and GGT levels in patients with normal and abnormal baseline FIB-4 index after 12 months of treatment. Cumulative normalization rates of ALT or GGT levels in **(a)** overall patients, **(b)** the male group and **(c)** the female group of normal baseline FIB-4 index with abnormal levels of both ALT and GGT; in **(d)** overall patients, **(e)** the male group and **(f)** the female group with abnormal ALT levels only; and in **(g)** overall patients, **(h)** the male group and **(i)** the female group with abnormal GGT levels only. Cumulative normalization rates of ALT or GGT levels in **(j)** overall patients, **(k)** the male group and **(l)** the female group of abnormal baseline FIB-4 index with abnormal levels of both ALT and GGT; in **(m)** overall patients, **(n)** the male group and **(o)** the female group with abnormal ALT levels only; and in **(p)** overall patients, **(q)** the male group and **(r)** the female group with abnormal GGT levels only.

**Additional file 7.** Correlations between GGT levels and the FIB-4 index. Scatter plots of the correlations between GGT levels and the FIB-4 index in **(a)** overall patients, **(b)** male patients and **(c)** female patients. Scatter plots of the correlations between GGT levels and FIB-4 index after 12 months of treatment in **(d)** overall patients, **(e)** male patients and **(f)** female patients. Scatter plots of the correlations between changed GGT levels and changed FIB-4 index after 12 months of treatment in **(g)** overall patients, **(h)** male patients and **(i)** female patients.

## Data Availability

The datasets used and/or analyzed during the current study are available from the corresponding author on reasonable request.

## Abbreviations

NAFLD: Nonalcoholic fatty liver disease
MAFLD: Metabolic associated fatty liver disease
MRI-PDFF: Magnetic resonance imaging-based proton density fat fraction
BMI: Body mass index
GGT: Gamma-glutamyl transferase
ALT: Alanine aminotransferase
OR: Odds ratio
CI: Confidence interval
HOMA-IR: Homeostasis model assessment of insulin resistance
CHOL: Cholesterol
TG: Triglyceride
T2DM: Type 2 diabetes mellitus
WHR: Waist-to-hip ratio
AST: Aspartate aminotransferase
ALP: Alkaline protease
TBil: Total bilirubin
DBil: Direct bilirubin
TBA: Total bile acid
LDH: Lactate dehydrogenase
CHE: Choline esterase
LAP: Leucine arylamidase
GLDH: Glutamate dehydrogenase
UA: Uric acid
HDL-C: High density lipoprotein-cholesterol
LDL-C: Low density lipoprotein-cholesterol
FFA: Free fatty acid
APOA: Apolipoprotein-A
APOB: Apolipoprotein-B
APOE: Apolipoprotein-E
LPA: Lipoprotein-a
FBG: Fasting blood glucose
FINS: Fasting insulin
IR: Insulin resistance
FIB-4: Fibrosis-4
SD: Standard deviations
IQR: Interquartile ranges
ROC: Receiver operating characteristic
AUC: Area under curve
LFC: Liver fat content
PFC: Pancreas fat content
ASFT: Abdominal subcutaneous fat thickness
