# Supplementary material for: Normalization of γ-glutamyl transferase levels is associated with better metabolic control in individuals with nonalcoholic fatty liver disease
Source: BMC Gastroenterol. 2021 May 10;21:215. doi: 10.1186/s12876-021-01790-w (PMC8112063; doi:10.1186/s12876-021-01790-w)
Supplement: Supplementary file 1 — Additional file 1. Summary of all additional tables (S1–S11). (Table S1) Medication status and follow-up time of NAFLD patients; Baseline characteristics of male (Table S2) and female (Table S3) NAFLD patients presenting different ALT and GGT statuses; Biochemical and metabolic changes from baseline to month 12 in male (Table S4) and female (Table S5) NAFLD patients presenting abnormal levels of both GGT and ALT at baseline; Factors associated with GGT normalization in overall patients (Table S6), male patients (Table S7) and female patients (Table S8) with NAFLD after 12 months of treatment predicted by the logistic regression model; Factors associated with ALT normalization in overall patients (Table S9), male patients (Table S10) and female patients (Table S11) with NAFLD after 12 months of treatment predicted by the logistic regression model. [file 12876_2021_1790_MOESM1_ESM.docx]

**Normalization of γ-glutamyl transferase levels is associated with better** **metabolic control in individuals with nonalcoholic fatty liver disease supplementary material**

**Table S1.** Medication status and follow-up time of NAFLD patients.

| Variables | Both GGT and ALT normalization (N = 53) | ALT normalization  only (N = 53) | GGT normalization  only (N = 73) | Both GGT and ALT abnormal (N = 307) | P |
| --- | --- | --- | --- | --- | --- |
| Follow up time, months | 10.0 (4.7,22.6) | 11.6 (3.9,34.5) | 11.4 (4.4,22.2) | 9.6 (4.9,17.7) | 0.12 |
| Male | 12.4 (4.8,21.3) | 10.8 (3.5,27.6) | 12.0 (5.6,22.7) | 9.7 (4.9,17.2) | 0.23 |
| Female | 9.7 (4.0,28.2) | 12.0 (4.8,37.3) | 9.0 (4.7,19.7) | 8.7 (4.9,19.0) | 0.50 |
| Anti-hyperlipidemic drug, n(%) | 18 (34.0) | 17 (32.1) | 23 (31.5) | 76 (24.8) | 0.33 |
| Statins | 7 (13.2) | 9 (17.0) | 11 (15.1) | 27 (8.8) |  |
| Orlistat | 8 (15.1) | 6 (11.3) | 7 (9.6) | 42 (13.7) |  |
| Fenofibrate | 2 (3.8) | 1 (1.9) | 2 (2.7) | 3 (1.0) |  |
| Ezetimibe | 1 (1.9) | 1 (1.9) | 3 (4.1) | 4 (1.3) |  |
| Anti-diabetes drug, n(%) | 4 (7.5) | 3 (5.7) | 7 (9.6) | 23 (7.5) | 0.87 |
| Metformin | 3 (5.7) | 2 (3.8) | 4 (5.5) | 15 (4.9) |  |
| Acarbose | 1 (1.9) | 1 (1.9) | 2 (2.7) | 5 (1.6) |  |
| Allopurinol | 0 (0.0) | 0 (0.0) | 1 (1.4) | 3 (1.0) |  |
| Uric acids lowering drug, n(%) | 4 (7.5) | 3 (5.7) | 5 (6.8) | 16 (5.2) | 0.88 |
| Benzbromarone | 2 (3.8) | 2 (3.8) | 3 (4.1) | 8 (2.6) |  |
| Allopurinol | 0 (0.0) | 0 (0.0) | 0 (0.0) | 1 (0.3) |  |
| Febuxostat | 2 (3.8) | 1 (1.9) | 2 (2.7) | 7 (2.3) |  |

Data are median (first quartile, third quartile) or n (%).

**Table S2.** Baseline characteristics of the male NAFLD patients presenting different ALT and GGT statuses.

| Variables | Both GGT and ALT abnormal (N = 370) | ALT abnormal only (N = 162) | GGT abnormal only (N = 126) | Both GGT and ALT normal  (N = 86) | P | Post-hoc | | | | | |  |
| --- | --- | --- | --- | --- | --- | --- | --- | --- | --- | --- | --- | --- |
|  |  |  |  |  |  | 1^#^ vs. 2^#^ | 1^#^ vs. 3^#^ | 1^#^ vs. 4^#^ | 2^#^ vs. 3^#^ | 2^#^ vs. 4^#^ | 3^#^ vs. 4^#^ |  |
| Age, years | 38.1 ± 11.3 | 39.8 ± 11.0 | 47.5 ± 11.5 | 46.7 ± 11.5 | < 0.001 | 0.10 | < 0.001 | < 0.001 | < 0.001 | < 0.001 | 0.72 |  |
| BMI, kg/m^2^ | 26.3 (24.5,28.7) | 25.9 (23.5,27.8) | 24.6 (22.4,26.7) | 25.4 (24.0,27.2) | < 0.001 | 0.94 | < 0.001 | < 0.001 | < 0.001 | < 0.001 | 0.20 |  |
| Waist-hip ratio | 0.90 (0.87,0.93) | 0.90 (0.87,0.93) | 0.90 (0.88,0.94) | 0.90 (0.87,0.93) | 0.18 |  |  |  |  |  |  |  |
| Smoke, n (%) | 90 (24.3) | 27 (16.7) | 43 (34.1) | 15 (17.4) | 0.01 | 0.05 | 0.03 | 0.17 | 0.001 | 0.88 | 0.01 |  |
| Complication, n (%) | | | | | | | | | | | |  |
| Type 2 diabetes | 21 (5.7) | 9 (5.6) | 3 (2.4) | 8 (9.3) | 0.19 |  |  |  |  |  |  |  |
| Hypertension | 112 (30.3) | 48 (29.6) | 37 (29.3) | 23 (26.7) | 0.94 |  |  |  |  |  |  |  |
| Liver biochemistry | | | | | | | | | | | |  |
| GGT, U/L | 96 (65,145) | 35 (29,42) | 83 (62,145) | 27 (20,35) | < 0.001 | < 0.001 | 0.16 | < 0.001 | 0.001 | 0.59 | < 0.001 |  |
| ALT, U/L | 68 (47,113) | 46 (38,59) | 24 (20,26) | 21 (18,26) | < 0.001 | < 0.001 | < 0.001 | < 0.001 | 0.04 | 0.001 | 0.97 |  |
| AST, U/L | 40 (30,60) | 31 (26,36) | 25 (20,33) | 21 (18,25) | < 0.001 | < 0.001 | 0.001 | < 0.001 | 0.45 | 0.06 | 0.63 |  |
| Alkaline phosphatase, U/L | 79 (68,90) | 75 (67,88) | 82 (73,94) | 71 (62,81) | < 0.001 | 0.001 | 0.10 | < 0.001 | 0.81 | 0.27 | 0.34 |  |
| Total bilirubin, umol/L | 13.8 (10.9,18.1) | 12.3 (10.2,19.7) | 10.8 (10.2,16.6) | 14.0 (10.9,18.2) | 0.20 |  |  |  |  |  |  |  |
| Direct bilirubin, umol/L | 2.7 (2.0,3.5) | 2.7 (1.9,3.6) | 2.8 (1.7,3.5) | 2.4 (2.0,3.2) | 0.03 | 0.07 | 0.05 | 0.02 | 0.39 | 0.62 | 0.60 |  |
| Total bile acid, umol/L | 2.9 (1.8,5.1) | 2.1 (1.1,2.8) | 2.8 (2.0,3.9) | 2.3 (1.6,3.5) | 0.14 |  |  |  |  |  |  |  |
| Lactate dehydrogenase, U/L | 197 (170,224) | 181 (166,211) | 168 (161,196) | 184 (165,204) | 0.02 | 0.05 | 0.02 | 0.01 | 0.34 | 0.71 | 0.49 |  |
| Choline esterase, U/L | 8948 (7876,10260) | 8976 (8127,10177) | 8197 (7653,9519) | 9084  (8174,9887) | 0.09 |  |  |  |  |  |  |  |
| Leucine arylamidase, U/L | 72 (64,89) | 60 (53,65) | 76 (63,88) | 56 (49,60) | < 0.001 | < 0.001 | 0.56 | < 0.001 | 0.04 | 0.44 | 0.01 |  |
| Glutamate dehydrogenase, U/L | 9.2 (5.4,14.0) | 5.3 (3.7,7.4) | 6.1 (4.6,7.6) | 3.1 (2.2,4.1) | < 0.001 | < 0.001 | 0.02 | < 0.001 | 0.36 | 0.04 | 0.02 |  |
| FIB-4 index | 0.74 (0.51,1.09) | 0.75 (0.54,1.05) | 1.14 (0.85,1.38) | 0.83 (0.59,1.16) | 0.79 |  |  |  |  |  |  |  |
| Metabolism | | | | | | | | | | | |  |
| Uric acid, umol/L | 443 (376,500) | 412 (314,498) | 413 (374,458) | 407 (349,469) | < 0.001 | 0.01 | 0.54 | < 0.001 | 0.37 | 0.14 | 0.06 |  |
| Hyperuricemia, n (%) | 225 (60.8) | 88 (54.3) | 71 (56.3) | 37 (43.0) | 0.02 | 0.16 | 0.38 | 0.01 | 0.73 | 0.09 | 0.06 |  |
| Cholesterol, mmol/L | 5.5 (4.9,6.1) | 5.1 (4.6,5.6) | 5.2 (4.4,6.2) | 5.3 (4.7,5.8) | 0.01 | 0.01 | 0.89 | 0.07 | 0.07 | 0.28 | 0.26 |  |
| Hyper-cholesterol,  n (%) | 164 (44.3) | 33 (20.4) | 55 (43.7) | 29 (33.7) | < 0.001 | < 0.001 | 0.89 | 0.07 | < 0.001 | 0.01 | 0.02 |  |
| Triglyceride, mmol/L | 2.0 (1.4,2.6) | 1.7 (1.3,2.0) | 2.4 (1.3,3.5) | 1.8 (1.1,2.4) | < 0.001 | 0.01 | 0.06 | 0.01 | 0.001 | 0.75 | 0.001 |  |
| Hyper-triglyceride,  n (%) | 238 (64.3) | 82 (50.6) | 83 (65.9) | 44 (51.2) | 0.01 | 0.01 | 0.75 | 0.02 | 0.01 | 0.94 | 0.03 |  |
|  |  |  |  |  |  |  |  |  |  |  |  |  |
| HDL-cholesterol, mmol/L | 1.1 (1.0,1.3) | 1.0 (1.0,1.2) | 1.1 (1.0,1.3) | 1.1 (1.0,1.3) | 0.31 |  |  |  |  |  |  |  |
| LDL-cholesterol, mmol/L | 3.6 (3.1,4.0) | 3.3 (3.1,4.0) | 3.1 (2.6,3.5) | 3.4 (2.9,3.8) | 0.21 |  |  |  |  |  |  |  |
| Free fatty acid, mmol/L | 570 (410,719) | 542 (444,705) | 572 (407,632) | 527 (433,606) | 0.11 |  |  |  |  |  |  |  |
| Apolipoprotein-A, mmol/L | 1.2 (1.1,1.4) | 1.2 (1.1,1.3) | 1.2 (1.2,1.4) | 1.3 (1.1,1.4) | 0.001 | 0.001 | 0.07 | 0.15 | < 0.001 | 0.10 | 0.01 |  |
| Apolipoprotein-B, mmol/L | 1.1 (0.9,1.2) | 1.0 (0.9,1.1) | 0.9 (0.8,1.1) | 1.0 (0.9,1.1) | 0.01 | 0.01 | 0.30 | 0.01 | 0.67 | 0.69 | 0.49 |  |
| Apolipoprotein-E, mmol/L | 47 (40,56) | 37 (33,46) | 47 (44,59) | 43 (34,49) | 0.01 | 0.01 | 0.75 | 0.01 | 0.2 | 0.79 | 0.27 |  |
| Lipoprotein-a, mmol/L | 105 (51,191) | 82 (38,275) | 77 (28,237) | 119 (53,252) | 0.14 |  |  |  |  |  |  |  |
| Fasting glucose, mmol/L | 5.0 (4.6,5.6) | 4.8 (4.5,5.2) | 5.0 (4.6,5.6) | 4.9 (4.6,5.6) | 0.68 |  |  |  |  |  |  |  |
| Fasting insulin, uU/mL | 9.2 (7.2,14.5) | 8.8 (6.5,12.6) | 7.6 (5.8,8.8) | 6.7 (4.9,9.6) | < 0.001 | 0.10 | 0.01 | < 0.001 | 0.09 | 0.001 | 0.60 |  |
| HOMA-IR | 2.0 (1.5,3.4) | 1.8 (1.3,2.6) | 1.5 (1.2,2.0) | 1.6 (1.1,2.3) | < 0.001 | 0.25 | 0.02 | < 0.001 | 0.12 | 0.01 | 0.72 |  |
| LFC †, % | 15.4 (9.2,22.4) | 15.9 (10.6,20.8) | 9.2 (6.8,15.8) | 8.7 (7.0,11.8) | < 0.001 | 0.95 | 0.01 | < 0.001 | 0.01 | < 0.001 | 0.38 |  |
| PFC ‡, % | 2.3 (1.6,3.7) | 2.2 (1.5,2.9) | 1.8 (1.6,3.5) | 2.3 (1.6,3.2) | 0.15 |  |  |  |  |  |  |  |
| ASFT ‖, mm | 20 (17,27) | 22 (18,27) | 20 (17,26) | 21 (17,25) | 0.50 |  |  |  |  |  |  |  |
| Medication | | | | | | | | | | | |  |
| Anti-hyperlipidemic drug, n (%) | 110 (29.7) | 38 (23.5) | 34 (26.9) | 26 (30.2) | 0.48 |  |  |  |  |  |  |  |
| Anti-Diabetes drug,  n (%) | 21 (5.7) | 9 (5.6) | 3 (2.4) | 5 (5.8) | 0.51 |  |  |  |  |  |  |  |
| Uric acid lowering drug, n (%) | 33 (8.9) | 20 (12.3) | 10 (7.9) | 9 (10.5) | 0.56 |  |  |  |  |  |  |  |

Data are median (first quartile, third quartile), n (%), or mean ± SD (standard deviation).

† mean N = 461; ‡ mean N = 459; ‖ mean N = 461.

# 1- both GGT and ALT abnormal group; 2- ALT abnormal only group; 3- GGT abnormal only group; 4- both GGT and ALT normal group.

Hyperuricemia was defined as uric acid level > 420 umol/L for male and > 360 umol/L for female; Hyper-cholesterol was defined as CHOL level > 5.7 mmol/L; Hyper-triglyceride was defined as TG level > 1.7 mmol/L.

**Table S3.** Baseline characteristics of the female NAFLD patients presenting different ALT and GGT statuses.

| Variables | Both GGT and ALT abnormal (N = 116) | ALT abnormal only (N = 119) | GGT abnormal only (N = 25) | Both GGT and ALT normal  (N = 44) | P | Post-hoc | | | | | |  |
| --- | --- | --- | --- | --- | --- | --- | --- | --- | --- | --- | --- | --- |
|  |  |  |  |  |  | 1^#^ vs. 2^#^ | 1^#^ vs. 3^#^ | 1^#^ vs. 4^#^ | 2^#^ vs. 3^#^ | 2^#^ vs. 4^#^ | 3^#^ vs. 4^#^ |  |
| Age, years | 47.7 ± 13.2 | 49.8 ± 14.1 | 51.1 ± 9.8 | 50.8 ± 11.5 | 0.37 |  |  |  |  |  |  |  |
| BMI, kg/m^2^ | 24.5 (22.8,26.2) | 24.2 (22.0,27.2) | 23.1 (20.4,25.8) | 23.4 (21.7,25.8) | 0.03 | 0.19 | 0.62 | 0.09 | 0.32 | 0.01 | 0.84 |  |
| Waist-hip ratio | 0.87 (0.85,0.91) | 0.86 (0.84,0.91) | 0.83 (0.81,0.85) | 0.84 (0.82,0.88) | 0.25 |  |  |  |  |  |  |  |
| Smoke, n (%) | 4 (3.4) | 2 (1.7) | 0 (0) | 1 (2.3) | 0.69 |  |  |  |  |  |  |  |
| Complication, n (%) | | | | | | | | | | | |  |
| Type 2 diabetes | 16 (13.8) | 13 (10.9) | 2 (8.0) | 5 (11.4) | 0.83 |  |  |  |  |  |  |  |
| Hypertension | 41 (35.3) | 41 (34.5) | 6 (24.0) | 13 (29.5) | 0.67 |  |  |  |  |  |  |  |
| Liver biochemistry | | | | | | | | | | | |  |
| GGT, U/L | 106 (69,167) | 30 (23,34) | 86 (78,94) | 20 (17,28) | < 0.001 | < 0.001 | 0.10 | < 0.001 | 0.16 | 0.66 | 0.12 |  |
| ALT, U/L | 70 (49,119) | 32 (26,41) | 16 (15,18) | 16 (12,18) | < 0.001 | < 0.001 | < 0.001 | < 0.001 | 0.30 | 0.01 | 0.96 |  |
| AST, U/L | 42 (34,100) | 30 (24,33) | 19 (18,20) | 19 (17,20) | < 0.001 | < 0.001 | 0.01 | < 0.001 | 0.55 | 0.11 | 0.97 |  |
| Alkaline phosphatase, U/L | 87 (78,116) | 74 (67,85) | 58 (46,71) | 72 (51,83) | < 0.001 | < 0.001 | 0.27 | < 0.001 | 0.58 | 0.82 | 0.52 |  |
| Total bilirubin, umol/L | 12.1 (9.0,14.6) | 10.3 (9.3,112.9) | 7.6 (6.0,9.1) | 10.1 (7.8,14.6) | 0.78 |  |  |  |  |  |  |  |
| Direct bilirubin, umol/L | 2.1 (1.6,3.3) | 1.9 (1.6,2.5) | 1.9 (1.7,2.0) | 1.6 (1.2,2.4) | 0.51 |  |  |  |  |  |  |  |
| Total bile acid, umol/L | 3.0 (2.0,6.2) | 2.2 (1.9,2.6) | 3.3 (1.7,3.9) | 2.3 (1.6,4.6) | 0.04 | 0.01 | 0.53 | 0.11 | 0.90 | 0.41 | 0.89 |  |
| Lactate dehydrogenase, U/L | 205 (185,219) | 210 (187,228) | 214 (179,250) | 188 (167,202) | 0.08 |  |  |  |  |  |  |  |
| Choline esterase, U/L | 8924 (8005,9729) | 8582 (7958,9669) | 7945 (5594,10296) | 9183 (8202,9962) | 0.68 |  |  |  |  |  |  |  |
| Leucine arylamidase, U/L | 82 (66,99) | 57 (49,61) | 81 (75,97) | 50 (45,54) | < 0.001 | < 0.001 | < 0.001 | < 0.001 | < 0.001 | 0.47 | < 0.001 |  |
| Glutamate dehydrogenase, U/L | 10.0 (4.8,13.0) | 4.4 (2.2,8.0) | 4.5 (3.8,5.2) | 2.6 (2.0,4.0) | < 0.001 | < 0.001 | 0.13 | < 0.001 | 0.94 | 0.46 | 0.88 |  |
| FIB-4 index | 1.08 (0.75,1.52) | 0.82 (0.61,1.26) | 0.84 (0.72,1.28) | 0.86 (0.66,1.12) | 0.10 |  |  |  |  |  |  |  |
| Metabolism | | | | | | | | | | | |  |
| Uric acid, umol/L | 377 (289,424) | 390 (335,429) | 311 (287,336) | 325 (290,422) | 0.06 |  |  |  |  |  |  |  |
| Hyperuricemia,  n (%) | 66 (56.9) | 54 (45.4) | 9 (36.0) | 15 (34.1) | 0.03 | 0.08 | 0.06 | 0.01 | 0.39 | 0.20 | 0.87 |  |
| Cholesterol, mmol/L | 5.3 (4.2,6.7) | 5.8 (5.1,6.7) | 4.8 (4.5,5.1) | 5.5 (4.9,6.4) | 0.11 |  |  |  |  |  |  |  |
| Hyper-cholesterol,  n (%) | 47 (40.5) | 54 (45.4) | 15 (60.0) | 25 (56.8) | 0.14 |  |  |  |  |  |  |  |
| Triglyceride, mmol/L | 1.7 (1.4,2.3) | 1.4 (1.1,1.9) | 1.2 (1.1,1.3) | 1.7 (1.5,2.7) | 0.81 |  |  |  |  |  |  |  |
| Hyper-triglyceride,  n (%) | 56 (48.3) | 52 (43.7) | 12 (48.0) | 18 (40.9) | 0.81 |  |  |  |  |  |  |  |
|  |  |  |  |  |  |  |  |  |  |  |  |  |
| HDL-cholesterol, mmol/L | 1.2 (1.0,1.4) | 1.3 (1.2,1.7) | 1.2 (1.0,1.3) | 1.3 (1.1,1.5) | 0.50 |  |  |  |  |  |  |  |
| LDL-cholesterol, mmol/L | 3.4 (2.7,4.2) | 3.7 (3.0,4.5) | 3.0 (2.8,3.2) | 3.5 (2.8,3.9) | 0.13 |  |  |  |  |  |  |  |
| Free fatty acid, mmol/L | 591 (479,731) | 472 (430,668) | 474 (428,521) | 487 (403,578) | 0.75 |  |  |  |  |  |  |  |
| Apolipoprotein-A, mmol/L | 1.3 (1.1,1.6) | 1.3 (1.2,1.5) | 1.5 (1.3,1.6) | 1.3 (1.1,1.6) | 0.79 |  |  |  |  |  |  |  |
| Apolipoprotein-B, mmol/L | 1.1 (0.8,1.2) | 1.1 (0.9,1.2) | 0.8 (0.7,0.9) | 1.1 (0.8,1.2) | 0.59 |  |  |  |  |  |  |  |
| Apolipoprotein-E, mmol/L | 49 (37,59) | 41 (35,49) | 42 (35,49) | 46 (36,68) | 0.92 |  |  |  |  |  |  |  |
| Lipoprotein-a, mmol/L | 130 (51,166) | 122 (80,282) | 269 (72,467) | 77 (58,382) | 0.26 |  |  |  |  |  |  |  |
| Fasting glucose, mmol/L | 5.0 (4.8,5.7) | 5.2 (4.6,5.7) | 5.1 (4.8,5.4) | 5.3 (4.7,6.2) | 0.42 |  |  |  |  |  |  |  |
| Fasting insulin, uU/mL | 9.5 (6.2,12.3) | 7.6 (6.0,10.5) | 9.1 (8.8,9.3) | 8.8 (6.3,10.2) | 0.03 | 0.57 | 0.40 | 0.01 | 0.49 | 0.02 | 0.90 |  |
| HOMA-IR | 2.2 (1.4,3.0) | 1.6 (1.2,2.4) | 2.0 (2.0,2.1) | 2.2 (1.7,2.5) | 0.14 |  |  |  |  |  |  |  |
| LFC †, % | 13.3 (8.6,25.8) | 15.1 (8.5,21.0) | 6.4 (6.4,7.3) | 10.8 (6.9,13.9) | 0.01 | 0.42 | 0.04 | 0.01 | 0.07 | 0.01 | 0.31 |  |
| PFC ‡, % | 2.5 (1.6,3.1) | 2.4 (1.7,3.6) | 2.0 (1.9,2.1) | 2.3 (1.3,3.2) | 0.14 |  |  |  |  |  |  |  |
| ASFT ‖, mm | 28 (22,31) | 30 (25,35) | 26 (25,26) | 28 (24,33) | 0.32 |  |  |  |  |  |  |  |
| Medication | | | | | | | | | | | |  |
| Anti-hyperlipidemic drug, n (%) | 36 (31.0) | 47 (39.5) | 14 (56.0) | 13 (29.5) | 0.07 |  |  |  |  |  |  |  |
| Anti-Diabetes drug, n (%) | 10 (8.6) | 8 (6.7) | 2 (8.0) | 3 (6.8) | 0.95 |  |  |  |  |  |  |  |
| Uric acid lowering drug, n (%) | 5 (4.3) | 4 (3.4) | 2 (8.0) | 1 (2.3) | 0.67 |  |  |  |  |  |  |  |

Data are median (first quartile, third quartile), n (%), or mean ± SD (standard deviation).

† mean N = 169; ‡ mean N = 169; ‖ mean N = 169.

# 1- both GGT and ALT abnormal group; 2- ALT abnormal only group; 3- GGT abnormal only group; 4- both GGT and ALT normal group.

Hyperuricemia was defined as uric acid level > 420 umol/L for male and > 360 umol/L for female; Hyper-cholesterol was defined as CHOL level > 5.7 mmol/L; Hyper-triglyceride was defined as TG level > 1.7 mmol/L.

**Table S4.** Biochemical and metabolic changes from baseline to month 12 in 370 male NAFLD patients presenting abnormal levels of both GGT and ALT at baseline.

| Variables | Both GGT and ALT normalization  (N = 42) | ALT normalization only (N = 43) | GGT normalization only (N = 52) | Both GGT and ALT abnormal  (N = 233) | P | Post-hoc | | | | | |
| --- | --- | --- | --- | --- | --- | --- | --- | --- | --- | --- | --- |
|  |  |  |  |  |  | 1^#^ vs. 2^#^ | 1^#^ vs. 3^#^ | 1^#^ vs. 4^#^ | 2^#^ vs. 3^#^ | 2^#^ vs. 4^#^ | 3^#^ vs. 4^#^ |
| Weight change, kg | -7.3 ± 13.4 | -0.1 ± 4.5 | -2.6 ± 4.4 | -0.4 ± 2.7 | < 0.001 | < 0.001 | 0.001 | < 0.001 | 0.07 | 0.74 | 0.03 |
| Weight change, % | -7.4 ± 10.2 | 0.1 ± 6.7 | -3.0 ± 5.1 | -0.5 ± 3.2 | < 0.001 | < 0.001 | 0.001 | < 0.001 | 0.02 | 0.60 | 0.01 |
| BMI, kg/m^2^ | -2.5 ± 4.9 | -0.1 ± 0.9 | -0.9 ± 1.5 | -0.1 ± 1.0 | < 0.001 | < 0.001 | 0.001 | < 0.001 | 0.14 | 0.95 | 0.04 |
| Waist-hip ratio | -0.04 ± 0.2 | -0.01 ± 0.1 | -0.01 ± 0.1 | -0.01 ± 0.2 | 0.02 | 0.03 | 0.02 | 0.01 | 0.94 | 0.73 | 0.79 |
| ALT, U/L | -57 (-70,-32) | -40 (-73,-24) | -51 (-87,-24) | -10 (-40,0) | 0.01 | 0.85 | 0.45 | 0.08 | 0.58 | 0.05 | 0.01 |
| AST, U/L | -17 (-25,-6) | -16 (-29,-3) | -14 (-29,-4) | 0 (-17,1) | 0.01 | 0.13 | 0.70 | 0.23 | 0.21 | 0.001 | 0.06 |
| GGT, U/L | -45 (-49,-30) | -28 (-88,-2) | -33 (-44,23) | -5 (-37,6) | 0.01 | 0.08 | 0.81 | 0.35 | 0.10 | 0.001 | 0.17 |
| ALP, U/L | -5 (-14,-1) | -1 (-14,5) | 0 (-14,6) | 0 (-3,4) | 0.13 |  |  |  |  |  |  |
| TBil, umol/L | -0.8 ± 6.0 | -4.9 ± 15.1 | 0.9 ± 5.7 | -0.4 ± 6.5 | 0.02 | 0.05 | 0.41 | 0.83 | 0.01 | 0.01 | 0.38 |
| DBil, umol/L | -0.1 ± 1.9 | -2.1 ± 7.0 | -0.1 ± 1.9 | -0.1 ± 1.9 | 0.01 | 0.01 | 0.94 | 0.98 | 0.01 | 0.001 | 0.90 |
| TBA, umol/L | -0.1 ± 3.1 | -1.1 ± 3.5 | 0.1 ± 3.5 | 0.1 ± 2.1 | 0.66 |  |  |  |  |  |  |
| LDH, U/L | -15.7 ± 48.7 | -11.7 ± 25.9 | -11.2 ± 35.1 | -6.3 ± 26.3 | 0.43 |  |  |  |  |  |  |
| CHE, U/L | -344 (-1602,-344) | -344 (-334,0) | -344 (-962,-344) | -344 (-334,-337) | 0.01 | < 0.001 | 0.06 | 0.01 | 0.05 | 0.10 | 0.37 |
| LAP, U/L | -3.7 ± 16.8 | -6.1 ± 14.4 | 0.8 ± 10.9 | -3.7 ± 15.9 | 0.31 |  |  |  |  |  |  |
| GLDH, U/L | -1.6 ± 5.6 | -0.7 ± 5.3 | -0.5 ± 3.7 | -0.6 ± 6.0 | 0.86 |  |  |  |  |  |  |
| FIB-4 index | 0.1 ± 1.4 | -0.2 ± 0.4 | -0.1 ± 0.3 | 0.1 ± 0.6 | 0.72 |  |  |  |  |  |  |
| UA, umol/L | -36 (-105,-15) | -20 (-89,0) | -21 (-76,0) | 0 (-24,0) | 0.11 |  |  |  |  |  |  |
| CHOL, mmol/L | -0.9 ± 1.6 | -0.3 ± 1.2 | -0.7 ± 1.4 | -0.3 ± 1.3 | 0.12 |  |  |  |  |  |  |
| TG, mmol/L | -0.6 ± 1.3 | -0.4 ± 0.8 | -0.4 ± 1.1 | -0.3 ± 1.0 | 0.40 |  |  |  |  |  |  |
| HDL-C, mmol/L | -0.1 ± 0.3 | -0.1 ± 0.2 | 0.1 ± 0.2 | -0.1 ± 0.3 | 0.48 |  |  |  |  |  |  |
| LDL-C, mmol/L | -0.6 ± 1.1 | -0.3 ± 0.8 | -0.2 ± 0.8 | -0.1 ± 0.9 | 0.05 |  |  |  |  |  |  |
| FFA, mmol/L | -56 (-212,0) | -25 (-72,0) | 0 (-56,0) | -47 (-56,0) | 0.07 |  |  |  |  |  |  |
| APOA, mmol/L | -0.1 ± 0.3 | 0.1 ± 0.3 | 0.1 ± 0.2 | -0.1 ± 0.2 | 0.38 |  |  |  |  |  |  |
| APOB, mmol/L | -0.1 ± 0.3 | -0.1 ± 0.2 | -0.1 ± 0.2 | -0.2 ± 0.2 | 0.83 |  |  |  |  |  |  |
| APOE, mmol/L | -8 (-15,-2) | -4 (-13,0) | 0 (-7,0) | 0 (-7,0) | 0.01 | 0.06 | 0.01 | 0.001 | 0.41 | 0.36 | 0.92 |
| LPA, mmol/L | 11 (0,64) | 11 (0,35) | 0 (0,16) | 0 (0,11) | 0.51 |  |  |  |  |  |  |
| FBG, mmol/L | -0.5 ± 1.7 | -0.3 ± 1.0 | -0.2 ± 0.5 | -0.1 ± 0.6 | 0.21 |  |  |  |  |  |  |
| FINS, uU/mL | -2.4 ± 4.8 | -1.4 ± 4.0 | -0.1 ± 3.2 | 0.1 ± 4.9 | 0.04 | 0.43 | 0.05 | 0.01 | 0.26 | 0.11 | 0.81 |
| HOMA-IR | -0.7 ± 1.3 | -0.5 ± 0.9 | -0.1 ± 0.8 | -0.1 ± 1.2 | 0.01 | 0.45 | 0.03 | 0.01 | 0.18 | 0.04 | 0.64 |

Data are median (first quartile, third quartile) or mean ± SD (standard deviation).

# 1- both GGT and ALT normalization group; 2- ALT normalization only group; 3- GGT normalization only group; 4- both GGT and ALT abnormal group.

**Table S5.** Biochemical and metabolic changes from baseline to month 12 in 116 female NAFLD patients presenting abnormal levels of both GGT and ALT at baseline.

| Variables | Both GGT and ALT normalization  (N = 11) | ALT normalization only (N = 10) | GGT normalization only (N = 21) | Both GGT and ALT abnormal  (N = 74) | P | Post-hoc | | | | | |
| --- | --- | --- | --- | --- | --- | --- | --- | --- | --- | --- | --- |
|  |  |  |  |  |  | 1^#^ vs. 2^#^ | 1^#^ vs. 3^#^ | 1^#^ vs. 4^#^ | 2^#^ vs. 3^#^ | 2^#^ vs. 4^#^ | 3^#^ vs. 4^#^ |
| Weight change, kg | -2.7 ± 3.7 | -0.1 ± 0.6 | -1.1 ± 2.6 | -0.7 ± 2.4 | 0.16 |  |  |  |  |  |  |
| Weight change, % | -3.9 ± 1.2 | -0.1 ± 0.5 | -1.6 ± 4.2 | -1.1 ± 3.6 | 0.23 |  |  |  |  |  |  |
| BMI, kg/m^2^ | -1.0 ± 1.4 | -0.1 ± 0.3 | -0.4 ± 1.1 | -0.3 ± 0.9 | 0.20 |  |  |  |  |  |  |
| Waist-hip ratio | -0.01 ± 0.1 | -0.01 ± 0.1 | 0.01 ± 0.1 | -0.01 ± 0.1 | 0.72 |  |  |  |  |  |  |
| ALT, U/L | -69 (-148,-43) | -46 (-52,-28) | -72 (-114,-28) | -9 (-60,9) | 0.42 |  |  |  |  |  |  |
| AST, U/L | -40 (-98,-24) | -11 (-29,-9) | -56 (-84,-13) | 0 (-16,8) | 0.15 |  |  |  |  |  |  |
| GGT, U/L | -60 (-90,-50) | -19 (-133,0) | -39 (-89,-17) | -6 (-50,5) | 0.54 |  |  |  |  |  |  |
| ALP, U/L | 0 (-5,12) | 0 (0,10) | -5 (-22,1) | 0 (-20,0) | 0.57 |  |  |  |  |  |  |
| TBil, umol/L | -0.8 ± 2.7 | -0.3 ± 0.8 | -0.9 ± 3.1 | -0.9 ± 10.4 | 0.99 |  |  |  |  |  |  |
| DBil, umol/L | -1.8 ± 4.6 | -0.1 ± 0.3 | -0.1 ± 1.8 | -0.8 ± 4.8 | 0.93 |  |  |  |  |  |  |
| TBA, umol/L | 0.6 ± 1.7 | 0.7 ± 3.2 | -0.9 ± 1.6 | 2.7 ± 7.3 | 0.41 |  |  |  |  |  |  |
| LDH, U/L | 10.2 ± 46.9 | 6.8 ± 16.0 | -14.2 ± 32.5 | -7.1 ± 35.7 | 0.33 |  |  |  |  |  |  |
| CHE, U/L | -180 (-180,679) | -180 (-180,275) | -180 (-180,0) | -180 (-180,0) | 0.44 |  |  |  |  |  |  |
| LAP, U/L | 4.4 ± 31.9 | -11.2 ± 19.7 | -11.5 ± 10.4 | -7.3 ± 18.7 | 0.29 |  |  |  |  |  |  |
| GLDH, U/L | 2.4 ± 10.6 | -3.6 ± 6.9 | -2.9 ± 4.0 | -2.6 ± 9.6 | 0.51 |  |  |  |  |  |  |
| FIB-4 index | 0.1 ± 0.6 | -0.1 ± 0.1 | 0.2 ± 0.6 | 0.1 ± 0.2 | 0.75 |  |  |  |  |  |  |
| UA, umol/L | 0 (-37,0) | 0 (-46,28) | -20 (-47,0) | 0 (-42,0) | 0.97 |  |  |  |  |  |  |
| CHOL, mmol/L | -1.3 ± 1.9 | 0.2 ± 0.5 | -0.5 ± 1.7 | -0.2 ± 1.1 | 0.13 |  |  |  |  |  |  |
| TG, mmol/L | -0.4 ± 0.7 | -0.1 ± 0.5 | -0.2 ± 0.6 | -0.2 ± 0.7 | 0.77 |  |  |  |  |  |  |
| HDL-C, mmol/L | -0.1 ± 0.3 | -0.1 ± 0.2 | 0.1 ± 0.6 | -0.1 ± 0.8 | 0.99 |  |  |  |  |  |  |
| LDL-C, mmol/L | -1.0 ± 1.6 | -0.1 ± 0.5 | -0.5 ± 0.9 | -0.1 ± 1.1 | 0.14 |  |  |  |  |  |  |
| FFA, mmol/L | 0 (-202,0) | 0 (0,0) | -53 (-150,0) | -53 (-53,0) | 0.02 | 0.01 | 0.03 | 0.01 | 0.36 | 0.65 | 0.42 |
| APOA, mmol/L | -0.1 ± 0.2 | 0.1 ± 0.1 | 0.1 ± 0.3 | -0.1 ± 0.3 | 0.05 |  |  |  |  |  |  |
| APOB, mmol/L | -0.1 ± 0.3 | -0.1 ± 0.1 | -0.1 ± 0.3 | -0.1 ± 0.2 | 0.32 |  |  |  |  |  |  |
| APOE, mmol/L | 0 (-8,0) | 0 (0,2) | -6 (-11,0) | 0 (-6,0) | 0.50 |  |  |  |  |  |  |
| LPA, mmol/L | 0 (-57,0) | 0 (-8,0) | -5 (-20,16) | 0 (-16,0) | 0.53 |  |  |  |  |  |  |
| FBG, mmol/L | -0.2 ± 0.4 | -0.1 ± 0.6 | -0.5 ± 0.7 | -0.2 ± 0.7 | 0.26 |  |  |  |  |  |  |
| FINS, uU/mL | -0.7 ± 2.6 | -0.5 ± 1.1 | -0.5 ± 4.0 | -0.4 ± 1.9 | 0.99 |  |  |  |  |  |  |
| HOMA-IR | -0.3 ± 0.7 | -0.5 ± 1.1 | -0.2 ± 1.1 | -0.2 ± 0.7 | 0.94 |  |  |  |  |  |  |

Data are median (first quartile, third quartile) or mean ± SD (standard deviation).

# 1- both GGT and ALT normalization group; 2- ALT normalization only group; 3- GGT normalization only group; 4- both GGT and ALT abnormal group.

**Table S6.** Factors associated with GGT normalization in patients with NAFLD after 12 months of treatment predicted by the logistic regression model.

| Factors | Overall cohort (N = 486) | | | | |  | Achieve ALT normalization subgroup (N = 106) | | | | |
| --- | --- | --- | --- | --- | --- | --- | --- | --- | --- | --- | --- |
|  | Univariate analysis | |  | Multivariable analysis ^b^ | |  | Univariate analysis | |  | Multivariable analysis ^b^ | |
|  | OR (95%CI) | P |  | OR (95%CI) | P |  | OR (95%CI) | P |  | OR (95%CI) | P |
| Baseline body weight, kg | 1.02 (1.00-1.04) | 0.04 |  | 1.01 (0.99-1.03) | 0.45 |  | 1.03 (0.99-1.06) | 0.12 |  |  |  |
| Weight loss, % | 1.24 (1.14-1.34) | < 0.001 |  | 1.21 (1.11-1.32) | < 0.001 |  | 1.44 (1.18-1.76) | < 0.001 |  | 1.43 (1.10-1.86) | 0.01 |
| Hypertension ^a^ | 1.29 (0.76-2.18) | 0.35 |  |  |  |  | 1.46 (0.54-3.94) | 0.45 |  |  |  |
| Type 2 diabetes | 1.06 (0.43-2.63) | 0.89 |  |  |  |  | 0.18 (0.02-1.59) | 0.12 |  |  |  |
| Baseline ALT, U/L | 1.00 (0.99-1.00) | 0.56 |  |  |  |  | 1.00 (0.99-1.01) | 0.83 |  |  |  |
| ALT decreased to normal ^c^ | 3.92 (2.22-6.91) | < 0.001 |  | 2.75 (1.41-5.36) | 0.01 |  | - | 1.00 |  |  |  |
| Baseline GGT, U/L | 0.99 (0.99-0.99) | 0.01 |  | 0.99 (0.98-0.99) | 0.01 |  | 0.98 (0.97-0.99) | 0.01 |  | 0.98 (0.96-0.99) | 0.01 |
| Change of TBil, umol/L | 0.98 (0.95-1.02) | 0.38 |  |  |  |  | 0.97 (0.91-1.03) | 0.26 |  |  |  |
| Change of DBil, umol/L | 0.99 (0.92-1.07) | 0.78 |  |  |  |  | 0.94 (0.82-1.07) | 0.35 |  |  |  |
| UA decreased to normal ^c^ | 1.39 (0.85-2.29) | 0.19 |  |  |  |  | 1.60 (0.62-4.18) | 0.33 |  |  |  |
| Baseline CHOL, mmol/L | 0.89 (0.75-1.06) | 0.21 |  |  |  |  | 1.20 (0.83-1.73) | 0.33 |  |  |  |
| CHOL decreased to normal ^c^ | 3.19 (1.64-6.21) | 0.01 |  | 2.41 (0.84-5.07) | 0.09 |  | 3.59 (1.01-12.73) | 0.04 |  | 2.79 (0.49-15.83) | 0.25 |
| Baseline TG, mmol/L | 0.81 (0.65-1.03) | 0.08 |  |  |  |  | 0.81 (0.52-1.28) | 0.37 |  |  |  |
| TG decreased to normal ^c^ | 2.49 (1.48-4.20) | 0.001 |  | 2.03 (1.11-3.71) | 0.02 |  | 2.19 (0.79-6.08) | 0.13 |  |  |  |
| Baseline HDL-C, mmol/L | 0.94 (0.57-1.53) | 0.80 |  |  |  |  | 6.37 (0.99-34.23) | 0.06 |  |  |  |
| Baseline LDL-C, mmol/L | 0.90 (0.70-1.16) | 0.43 |  |  |  |  | 1.30 (0.80-2.10) | 0.29 |  |  |  |
| LDL-C decreased to normal ^c^ | 1.58 (0.95-2.64) | 0.08 |  |  |  |  | 1.13 (0.43-2.99) | 0.80 |  |  |  |
| FBG decreased to normal ^c^ | 3.14 (1.20-8.23) | 0.02 |  | 2.10 (0.72-6.13) | 0.17 |  | 2.76 (0.50-15.33) | 0.25 |  |  |  |
| Baseline FINS, uU/mL | 0.97 (0.93-0.99) | 0.04 |  | 0.97 (0.92-1.03) | 0.38 |  | 0.93 (0.84-1.03) | 0.14 |  |  |  |
| FINS decreased to normal ^c^ | 1.92 (0.55-6.75) | 0.31 |  |  |  |  | 1.84 (0.73-1.96) | 0.14 |  |  |  |
| HOMA-IR decreased to normal ^c^ | 2.16 (1.26-3.68) | 0.01 |  | 2.04 (1.07-3.89) | 0.03 |  | 6.52 (2.04-20.89) | 0.01 |  | 5.01 (1.09-23.08) | 0.04 |

^a^ Hypertension was defined as systolic blood pressure (SBP) ≥ 140 mmHg or diastolic blood pressure (DBP) ≥ 90 mmHg; ^b^ Multivariable analysis would be further adjusted by smoke, and the variate of smoke was categorized according to the number of cigarettes as 0 (never smoker), 1-9, 10-19, 20-39 and over 40; ^c^ ALT decreased to normal was defined as ALT level ≤ 30 U/L in male and ALT level ≤ 19 U/L in female; UA decreased to normal was defined as uric acid level ≤ 420 umol/L for male and ≤ 360 umol/L for female; CHOL decreased to normal was defined as CHOL level ≤ 5.7 mmol/L; TG decreased to normal was defined as TG level ≤ 1.7 mmol/L; LDL-C decreased to normal was defined as LDL-C level ≤ 3.4 mmol/L; FBG decreased to normal was defined as FBG level ≤ 6 mmol/L; FINS decreased to normal was defined as FINS level ≤ 23 uU/mL; HOMA-IR decreased to normal was defined as HOMA-IR level ≤ 2.69.

**Table S7.** Factors associated with GGT normalization in the male NAFLD patients after 12 months of treatment.

| Factors | Overall cohort (N = 370) | | | | |  | Achieve ALT normalization subgroup (N = 85) | | | | |
| --- | --- | --- | --- | --- | --- | --- | --- | --- | --- | --- | --- |
|  | Univariate analysis | |  | Multivariable analysis ^b^ | |  | Univariate analysis | |  | Multivariable analysis ^b^ | |
|  | OR (95%CI) | P |  | OR (95%CI) | P |  | OR (95%CI) | P |  | OR (95%CI) | P |
| Baseline body weight, kg | 1.02 (1.00-1.04) | 0.03 |  | 1.02 (0.99-1.05) | 0.28 |  | 1.03 (0.99-1.07) | 0.16 |  |  |  |
| Weight loss, % | 1.31 (1.19-1.45) | < 0.001 |  | 1.22 (1.09-1.37) | 0.001 |  | 1.45 (1.16-1.82) | 0.001 |  | 1.51 (1.11-2.06) | 0.01 |
| Hypertension ^a^ | 1.52 (0.82-2.80) | 0.19 |  |  |  |  | 1.18 (0.39-3.60) | 0.78 |  |  |  |
| Type 2 diabetes | 0.80 (0.22-2.97) | 0.74 |  |  |  |  | 0.31 (0.03-3.16) | 0.32 |  |  |  |
| Baseline ALT, U/L | 1.00 (0.99-1.00) | 0.57 |  |  |  |  | 0.99 (0.99-1.01) | 0.66 |  |  |  |
| ALT decreased to normal ^c^ | 4.49 (2.35-8.57) | < 0.001 |  | 3.43 (1.66-7.07) | 0.01 |  | - | - |  |  |  |
| Baseline GGT, U/L | 0.99 (0.98-0.99) | 0.01 |  | 0.99 (0.98-0.99) | 0.01 |  | 0.98 (0.97-0.99) | 0.02 |  | 0.97 (0.95-0.99) | 0.01 |
| Change of TBil, umol/L | 0.98 (0.93-1.02) | 0.29 |  |  |  |  | 0.96 (0.90-1.03) | 0.22 |  |  |  |
| Change of DBil, umol/L | 0.95 (0.83-1.08) | 0.45 |  |  |  |  | 0.87 (0.68-1.10) | 0.25 |  |  |  |
| UA decreased to normal ^c^ | 1.46 (0.82-2.60) | 0.20 |  |  |  |  | 1.00 (0.99-1.01) | 0.58 |  |  |  |
| Baseline CHOL, mmol/L | 0.92 (0.75-1.11) | 0.37 |  |  |  |  | 1.22 (0.81-1.85) | 0.35 |  |  |  |
| CHOL decreased to normal ^c^ | 2.77 (1.32-5.82) | 0.02 |  | 2.33 (0.73-4.22) | 0.15 |  | 1.31 (0.31-5.51) | 0.72 |  |  |  |
| Baseline TG, mmol/L | 0.83 (0.65-1.07) | 0.15 |  |  |  |  | 0.87 (0.54-1.39) | 0.55 |  |  |  |
| TG decreased to normal ^a^ | 2.54 (1.39-4.63) | 0.01 |  | 2.31 (1.19-4.50) | 0.02 |  | 1.63 (0.53-5.04) | 0.39 |  |  |  |
| Baseline HDL-C, mmol/L | 0.91 (0.44-1.85) | 0.79 |  |  |  |  | 7.20 (0.99-52.48) | 0.05 |  |  |  |
| Baseline LDL-C, mmol/L | 0.87 (0.65-1.17) | 0.37 |  |  |  |  | 1.37 (0.79-2.40) | 0.26 |  |  |  |
| LDL-C decreased to normal ^c^ | 1.33 (0.74-2.38) | 0.34 |  |  |  |  | 0.74 (0.25-2.19) | 0.58 |  |  |  |
| FBG decreased to normal ^c^ | 1.88 (1.59-4.07) | 0.02 |  | 1.70 (0.64-3.83) | 0.07 |  | 3.25 (0.32-33.41) | 0.32 |  |  |  |
| Baseline FINS, uU/mL | 0.95 (0.89-1.01) | 0.08 |  |  |  |  | 0.94 (0.85-1.04) | 0.24 |  |  |  |
| HOMA-IR decreased to normal ^c^ | 3.18 (1.71-5.91) | < 0.001 |  | 2.65 (1.34-5.24) | 0.01 |  | 3.65 (1.87-15.56) | 0.01 |  | 7.87 (0.99-62.59) | 0.05 |

^a^ Hypertension was defined as systolic blood pressure (SBP) ≥ 140 mmHg or diastolic blood pressure (DBP) ≥ 90 mmHg; ^b^ Multivariable analysis would be further adjusted by smoke, and the variate of smoke was categorized according to the number of cigarettes as 0 (never smoker), 1-9, 10-19, 20-39 and over 40; ^c^ ALT decreased to normal was defined as ALT level ≤ 30 U/L in male and ALT level ≤ 19 U/L in female; UA decreased to normal was defined as uric acid level ≤ 420 umol/L for male and ≤ 360 umol/L for female; CHOL decreased to normal was defined as CHOL level ≤ 5.7 mmol/L; TG decreased to normal was defined as TG level ≤ 1.7 mmol/L; LDL-C decreased to normal was defined as LDL-C level ≤ 3.4 mmol/L; FBG decreased to normal was defined as FBG level ≤ 6 mmol/L; HOMA-IR decreased to normal was defined as HOMA-IR level ≤ 2.69.

**Table S8.** Factors associated with GGT normalization in the female NAFLD patients after 12 months of treatment.

| Factors | Overall cohort (N = 116) | | | | | | | | |  | | Achieve ALT normalization subgroup (N = 21) | | | | | | | | | |  |  |
| --- | --- | --- | --- | --- | --- | --- | --- | --- | --- | --- | --- | --- | --- | --- | --- | --- | --- | --- | --- | --- | --- | --- | --- |
|  | Univariate analysis | | |  | | Multivariable analysis ^b^ | | | |  | | Univariate analysis | | | |  | | Multivariable analysis ^b^ | | | |  |  |
|  | OR (95%CI) | P | |  | | OR (95%CI) | | P | |  | | OR (95%CI) | | P | |  | | OR (95%CI) | | P | |  |  |
| Baseline body weight, kg | 1.03 (0.98-1.07) | | 0.20 | |  | |  | |  | |  | | 1.03 (0.96-1.12) | | 0.40 | |  | |  | |  | |  |
| Weight loss, % | 1.10 (0.97-1.24) | | 0.16 | |  | |  | |  | |  | | 1.69 (0.80-3.56) | | 0.17 | |  | |  | |  | |  |
| Hypertension ^a^ | 0.81 (0.29-2.29) | | 0.69 | |  | |  | |  | |  | | 3.33 (0.36-30.70) | | 0.29 | |  | |  | |  | |  |
| Type 2 diabetes | 1.37 (0.37-5.07) | | 0.64 | |  | |  | |  | |  | | - | | 0.98 | |  | |  | |  | | |
| Baseline ALT, U/L | 1.00 (0.99-1.01) | | 0.87 | |  | |  | |  | |  | | 1.02 (0.99-1.04) | | 0.20 | |  | |  | |  | | |
| ALT decreased to normal ^c^ | 2 67 (0.79-9.05) | | 0.11 | |  | |  | |  | |  | | - | | - | |  | |  | |  | | |
| Baseline GGT, U/L | 0.99 (0.99-1.00) | | 0.20 | |  | |  | |  | |  | | 0.99 (0.97-1.01) | | 0.19 | |  | |  | |  | | |
| Change of TBil, umol/L | 1.00 (0.95-1.06) | | 0.98 | |  | |  | |  | |  | | 1.39 (0.73-2.65) | | 0.31 | |  | |  | |  | | |
| Change of DBil, umol/L | 1.02 (0.93-1.11) | | 0.73 | |  | |  | |  | |  | | 1.36 (0.60-3.07) | | 0.46 | |  | |  | |  | | |
| UA decreased to normal ^c^ | 1.17 (0.43-3.18) | | 0.77 | |  | |  | |  | |  | | 0.97 (0.94-1.01) | | 0.06 | |  | |  | |  | | |
| Baseline CHOL, mmol/L | 0.83 (0.57-1.20) | | 0.32 | |  | |  | |  | |  | | 1.13 (0.49-2.58) | | 0.78 | |  | |  | |  | | |
| CHOL decreased to normal ^c^ | 5.25 (1.12-24.65) | | 0.04 | |  | | 5.25 (1.12-24.65) | | 0.04 | |  | | 2.40 (0.17-34.92) | | 0.52 | |  | |  | |  | | |
| Baseline TG, mmol/L | 0.74 (0.40-1.36) | | 0.33 | |  | |  | |  | |  | | 0.30 (0.04-2.02) | | 0.21 | |  | |  | |  | | |
| TG decreased to normal ^c^ | 2.32 (0.80-6.70) | | 0.12 | |  | |  | |  | |  | | 8.00 (0.60-106.94) | | 0.12 | |  | |  | |  | | |
| Baseline HDL-C, mmol/L | 0.92 (0.45-1.88) | | 0.82 | |  | |  | |  | |  | | 5.73 (0.20-167.00) | | 0.31 | |  | |  | |  | | |
| Baseline LDL-C, mmol/L | 1.01 (0.61-1.66) | | 0.98 | |  | |  | |  | |  | | 1.07 (0.38-3.00) | | 0.89 | |  | |  | |  | | |
| LDL-C decreased to normal ^c^ | 2.75 (0.90-8.39) | | 0.08 | |  | |  | |  | |  | | 8.00 (0.60-106.94) | | 0.12 | |  | |  | |  | | |
| FBG decreased to normal ^c^ | 0.95 (0.27-3.39) | | 0.94 | |  | |  | |  | |  | | 2.40 (0.17-34.93) | | 0.52 | |  | |  | |  | | |
| Baseline FINS, uU/mL | 1.02 (0.94-1.10) | | 0.71 | |  | |  | |  | |  | | 0.84 (0.62-1.16) | | 0.30 | |  | |  | |  | | |
| FINS decreased to normal ^c^ | 0.35 (0.07-1.88) | | 0.22 | |  | |  | |  | |  | | 0.63 (0.22-1.75) | | 0.37 | |  | |  | |  | | |
| HOMA-IR decreased to normal ^c^ | 0.57 (0.19-1.70) | | 0.31 | |  | |  | |  | |  | | 0.45 (0.10-2.06) | | 0.31 | |  | |  | |  | | |

^a^ Hypertension was defined as systolic blood pressure (SBP) ≥ 140 mmHg or diastolic blood pressure (DBP) ≥ 90 mmHg; ^b^ Multivariable analysis would be further adjusted by smoke, and the variate of smoke was categorized according to the number of cigarettes as 0 (never smoker), 1-9, 10-19, 20-39 and over 40; ^c^ ALT decreased to normal was defined as ALT level ≤ 30 U/L in male and ALT level ≤ 19 U/L in female; UA decreased to normal was defined as uric acid level ≤ 420 umol/L for male and ≤ 360 umol/L for female; CHOL decreased to normal was defined as CHOL level ≤ 5.7 mmol/L; TG decreased to normal was defined as TG level ≤ 1.7 mmol/L; LDL-C decreased to normal was defined as LDL-C level ≤ 3.4 mmol/L; FBG decreased to normal was defined as FBG level ≤ 6 mmol/L; HOMA-IR decreased to normal was defined as HOMA-IR level ≤ 2.69.

**Table S9.** Factors associated with ALT normalization in patients with NAFLD after 12 months of treatment predicted by the logistic regression model.

| Factors | Overall cohort (N = 486) | | | | |  | Achieve GGT normalization subgroup (N = 126) | | | | |
| --- | --- | --- | --- | --- | --- | --- | --- | --- | --- | --- | --- |
|  | Univariate analysis | |  | Multivariable analysis ^b^ | |  | Univariate analysis | |  | Multivariable analysis ^b^ | |
|  | OR (95%CI) | P |  | Odds ratio | P |  | OR (95%CI) | P |  | OR (95%CI) | P |
| Baseline body weight, kg | 1.00 (0.99-1.02) | 0.76 |  |  |  |  | 1.00 (0.97-1.03) | 0.90 |  |  |  |
| Weight loss, % | 1.09 (1.02-1.16) | 0.01 |  | 1.03 (0.97-1.10) | 0.31 |  | 1.11 (1.01-1.22) | 0.03 |  | 1.08 (0.97-1.19) | 0.16 |
| Hypertension ^a^ | 1.39 (0.79-2.45) | 0.25 |  |  |  |  | 1.46 (0.59-3.65) | 0.42 |  |  |  |
| Type 2 diabetes | 1.19 (0.46-3.09) | 0.72 |  |  |  |  | 0.24 (0.03-2.09) | 0.20 |  |  |  |
| Baseline ALT, U/L | 0.99 (0.99-0.99) | 0.03 |  | 0.99 (0.99-0.99) | 0.03 |  | 0.99 (0.98-0.99) | 0.02 |  | 0.99 (0.98-0.99) | 0.04 |
| Baseline GGT, U/L | 1.00 (0.99-1.00) | 0.81 |  |  |  |  | 0.99 (0.99-1.00) | 0.42 |  |  |  |
| GGT decreased to normal ^c^ | 3.92 (2.22-6.91) | < 0.001 |  | 2.99 (1.55-5.77) | 0.001 |  | - | - |  |  |  |
| Change of TBil, umol/L | 1.03 (0.99-1.06) | 0.09 |  |  |  |  | 1.04 (0.96-1.13) | 0.35 |  |  |  |
| Change of DBil, umol/L | 1.05 (0.98-1.13) | 0.13 |  |  |  |  | 1.05 (0.86-1.27) | 0.63 |  |  |  |
| UA decreased to normal ^c^ | 1.13 (0.66-1.94) | 0.65 |  |  |  |  | 1.11 (0.46-2.67) | 0.81 |  |  |  |
| Baseline CHOL, mmol/L | 0.93 (0.77-1.12) | 0.44 |  |  |  |  | 1.21 (0.88-1.66) | 0.25 |  |  |  |
| CHOL decreased to normal ^c^ | 2.16 (1.10-4.25) | 0.03 |  | 1.44 (0.69-3.02) | 0.33 |  | 0.87 (0.25-3.01) | 0.83 |  |  |  |
| Baseline TG, mmol/L | 0.85 (0.66-1.09) | 0.19 |  |  |  |  | 0.89 (0.61-1.31) | 0.55 |  |  |  |
| TG decreased to normal ^c^ | 1.88 (1.08-3.27) | 0.03 |  | 1.38 (0.74-2.56) | 0.31 |  | 1.41 (0.54-3.67) | 0.48 |  |  |  |
| Baseline HDL-C, mmol/L | 0.97 (0.58-1.63) | 0.91 |  |  |  |  | 3.28 (0.94-11.47) | 0.06 |  |  |  |
| Baseline LDL-C, mmol/L | 1.05 (0.80-1.37) | 0.75 |  |  |  |  | 1.56 (0.94-2.56) | 0.08 |  |  |  |
| LDL-C decreased to normal ^c^ | 1.18 (0.68-2.03) | 0.56 |  |  |  |  | 0.75 (0.30-1.85) | 0.53 |  |  |  |
| FBG decreased to normal ^c^ | 1.45 (0.62-3.42) | 0.39 |  |  |  |  | 0.95 (0.15-6.00) | 0.96 |  |  |  |
| Baseline FINS, uU/mL | 0.94 (0.88-0.99) | 0.02 |  | 1.00 (0.94-1.07) | 0.96 |  | 0.90 (0.83-0.99) | 0.03 |  | 0.95 (0.83-1.09) | 0.45 |
| FINS decreased to normal ^c^ | 2.21 (0.54-6.40) | 0.99 |  |  |  |  | 1.87 (0.97-3.94) | 0.99 |  |  |  |
| HOMA-IR decreased to normal ^c^ | 3.26 (1.75-6.08) | < 0.001 |  | 3.01 (1.32-6.84) | 0.01 |  | 7.25 (2.23-23.55) | 0.01 |  | 5.50 (1.59-18.98) | 0.01 |

^a^ Hypertension was defined as systolic blood pressure (SBP) ≥ 140 mmHg or diastolic blood pressure (DBP) ≥ 90 mmHg; ^b^ Multivariable analysis would be further adjusted by smoke, and the variate of smoke was categorized according to the number of cigarettes as 0 (never smoker), 1-9, 10-19, 20-39 and over 40; GGT decreased to normal was defined as GGT level ≤ 50 U/L; UA decreased to normal was defined as uric acid level ≤ 420 umol/L for male and ≤ 360 umol/L for female; CHOL decreased to normal was defined as CHOL level ≤ 5.7 mmol/L; TG decreased to normal was defined as TG level ≤ 1.7 mmol/L; LDL-C decreased to normal was defined as LDL-C level ≤ 3.4 mmol/L; FBG decreased to normal was defined as FBG level ≤ 6 mmol/L; FINS decreased to normal was defined as FINS level ≤ 23 uU/mL; HOMA-IR decreased to normal was defined as HOMA-IR level ≤ 2.69.

**Table S10.** Factors associated with ALT normalization in the male NAFLD patients after 12 months of treatment.

| Factors | Overall cohort (N = 370) | | | | |  | Achieve GGT normalization subgroup (N = 94) | | | | |
| --- | --- | --- | --- | --- | --- | --- | --- | --- | --- | --- | --- |
|  | Univariate analysis | |  | Multivariable analysis ^b^ | |  | Univariate analysis | |  | Multivariable analysis ^b^ | |
|  | OR (95%CI) | P |  | Odds ratio | P |  | OR (95%CI) | P |  | OR (95%CI) | P |
| Baseline body weight, kg | 0.99 (0.98-1.02) | 0.77 |  |  |  |  | 0.99 (0.96-1.03) | 0.58 |  |  |  |
| Weight loss, % | 1.10 (1.03-1.19) | 0.01 |  | 1.04 (0.97-1.11) | 0.29 |  | 1.12 (0.99-1.25) | 0.05 |  |  |  |
| Hypertension ^a^ | 1.46 (0.77-2.78) | 0.25 |  |  |  |  | 1.13 (0.40-3.21) | 0.82 |  |  |  |
| Type 2 diabetes | 1.46 (0.44-4.84) | 0.54 |  |  |  |  | 0.64 (0.05-7.39) | 0.72 |  |  |  |
| Baseline ALT, U/L | 0.99 (0.99-1.00) | 0.07 |  |  |  |  | 0.99 (0.98-0.99) | 0.03 |  | 0.98 (0.97-0.99) | 0.01 |
| Baseline GGT, U/L | 0.99 (0.99-1.00) | 0.07 |  |  |  |  | 0.77 (0.26-1.03) | 0.31 |  |  |  |
| GGT decreased to normal ^c^ | 4.49 (2.35-8.57) | < 0.001 |  | 2.99 (1.41-6.37) | 0.01 |  | - | - |  |  |  |
| Change of TBil, umol/L | 1.04 (1.00-1.08) | 0.04 |  | 1.02 (0.85-1.48) | 0.12 |  | 1.05 (0.96-1.15) | 0.28 |  |  |  |
| Change of DBil, umol/L | 1.11 (0.99-1.23) | 0.08 |  |  |  |  | 1.02 (0.78-1.33) | 0.91 |  |  |  |
| UA decreased to normal ^c^ | 1.17 (0.64-2.15) | 0.62 |  |  |  |  | 0.76 (0.28-2.07) | 0.59 |  |  |  |
| Baseline CHOL, mmol/L | 0.94 (0.77-1.15) | 0.54 |  |  |  |  | 1.23 (0.84-1.80) | 0.29 |  |  |  |
| CHOL decreased to normal ^c^ | 2.56 (1.18-5.59) | 0.02 |  | 1.89 (0.82-4.36) | 0.13 |  | 1.19 (0.30-4.72) | 0.81 |  |  |  |
| Baseline TG, mmol/L | 0.86 (0.67-1.11) | 0.25 |  |  |  |  | 0.93 (0.62-1.40) | 0.73 |  |  |  |
| TG decreased to normal ^c^ | 2.27 (1.21-4.25) | 0.01 |  | 1.69 (0.84-3.38) | 0.14 |  | 1.40 (0.48-4.11) | 0.54 |  |  |  |
| Baseline HDL-C, mmol/L | 1.14 (0.58-2.24) | 0.70 |  |  |  |  | 24.44 (2.02-295.56) | 0.01 |  | 50.14 (0.97-261.38) | 0.05 |
| Baseline LDL-C, mmol/L | 1.08 (0.79-1.46) | 0.63 |  |  |  |  | 1.87 (1.01-3.47) | 0.04 |  | 4.76 (1.49-15.21) | 0.01 |
| LDL-C decreased to normal ^c^ | 1.28 (0.70-1.37) | 0.42 |  |  |  |  | 0.74 (0.27-2.05) | 0.56 |  |  |  |
| FBG decreased to normal ^c^ | 2.05 (0.68-6.13) | 0.20 |  |  |  |  | 0.81 (0.33-1.97) | 0.64 |  |  |  |
| Baseline FINS, uU/mL | 0.94 (0.88-0.99) | 0.04 |  | 0.97 (0.89-1.04) | 0.37 |  | 0.92 (0.83-1.02) | 0.12 |  |  |  |
| HOMA-IR decreased to normal ^c^ | 3.75 (1.92-7.35) | < 0.001 |  | 1.95 (1.02-4.47) | 0.01 |  | 6.03 (1.95-23.90) | 0.01 |  | 10.50 (1.88-58.68) | 0.01 |

^a^ Hypertension was defined as systolic blood pressure (SBP) ≥ 140 mmHg or diastolic blood pressure (DBP) ≥ 90 mmHg; ^b^ Multivariable analysis would be further adjusted by smoke, and the variate of smoke was categorized according to the number of cigarettes as 0 (never smoker), 1-9, 10-19, 20-39 and over 40; ^c^ GGT decreased to normal was defined as GGT level ≤ 50 U/L; UA decreased to normal was defined as uric acid level ≤ 420 umol/L for male and ≤ 360 umol/L for female; CHOL decreased to normal was defined as CHOL level ≤ 5.7 mmol/L; TG decreased to normal was defined as TG level ≤ 1.7 mmol/L; LDL-C decreased to normal was defined as LDL-C level ≤ 3.4 mmol/L; FBG decreased to normal was defined as FBG level ≤ 6 mmol/L; HOMA-IR decreased to normal was defined as HOMA-IR level ≤ 2.69.

**Table S11.** Factors associated with ALT normalization in the female NAFLD patients after 12 months of treatment.

| Factors | Overall cohort (N = 116) | | | | |  | Achieve GGT normalization subgroup (N = 32) | | | | |
| --- | --- | --- | --- | --- | --- | --- | --- | --- | --- | --- | --- |
|  | Univariate analysis | |  | Multivariable analysis ^b^ | |  | Univariate analysis | |  | Multivariable analysis ^b^ | |
|  | OR (95%CI) | P |  | Odds ratio | P |  | OR (95%CI) | P |  | OR (95%CI) | P |
| Baseline body weight, kg | 1.00 (0.95-1.06) | 0.94 |  |  |  |  | 0.99 (0.92-1.07) | 0.77 |  |  |  |
| Weight loss, % | 1.02 (0.88-1.19) | 0.79 |  |  |  |  | 1.08 (0.88-1.32) | 0.47 |  |  |  |
| Hypertension ^a^ | 1.28 (0.38-4.34) | 0.70 |  |  |  |  | 3.25 (0.46-22.93) | 0.24 |  |  |  |
| Type 2 diabetes | 1.09 (0.21-5.67) | 0.92 |  |  |  |  | - | 0.99 |  |  |  |
| Baseline ALT, U/L | 0.99 (0.98-1.00) | 0.18 |  |  |  |  | 0.99 (0.97-1.01) | 0.32 |  |  |  |
| Baseline GGT, U/L | 0.99 (0.99-1.00) | 0.47 |  |  |  |  | - | - |  |  |  |
| GGT decreased to normal ^c^ | 2.67 (0.79-9.05) | 0.11 |  |  |  |  | 0.99 (0.98-1.01) | 0.60 |  |  |  |
| Change of TBil, umol/L | 0.99 (0.90-1.08) | 0.79 |  |  |  |  | 1.00 (0.73-1,38) | 0.98 |  |  |  |
| Change of DBil, umol/L | 1.01 (0.91-1.13) | 0.82 |  |  |  |  | 1.19 (0.86-1.63) | 0.30 |  |  |  |
| UA decreased to normal ^c^ | 1.41 (0.40-5.04) | 0.59 |  |  |  |  | - | 0.99 |  |  |  |
| Baseline CHOL, mmol/L | 0.84 (0.53-1.34) | 0.47 |  |  |  |  | 1.07 (0.55-2.09) | 0.84 |  |  |  |
| CHOL decreased to normal ^c^ | 1.24 (0.31-5.00) | 0.76 |  |  |  |  | 0.31 (0.02-5.96) | 0.44 |  |  |  |
| Baseline TG, mmol/L | 0.58 (0.25-1.37) | 0.21 |  |  |  |  | 0.33 (0.06-1.71) | 0.19 |  |  |  |
| TG decreased to normal ^c^ | 1.07 (0.32-3.60) | 0.92 |  |  |  |  | 2.08 (0.19-22.67) | 0.55 |  |  |  |
| Baseline HDL-C, mmol/L | 0.89 (0.35-2.29) | 0.81 |  |  |  |  | 1.73 (0.36-8.24) | 0.49 |  |  |  |
| Baseline LDL-C, mmol/L | 0.88 (0.48-1.64) | 0.70 |  |  |  |  | 0.82 (0.27-2.46) | 0.72 |  |  |  |
| LDL-C decreased to normal ^c^ | 0.95 (0.28-3.20) | 0.93 |  |  |  |  | 1.54 (0.14-17.33) | 0.73 |  |  |  |
| FBG decreased to normal ^c^ | 0.62 (0.15-2.63) | 0.52 |  |  |  |  | 1.07 (0.09-12.83) | 0.96 |  |  |  |
| Baseline FINS, uU/mL | 0.90 (0.77-1.04) | 0.16 |  |  |  |  | 0.82 (0.64-1.06) | 0.13 |  |  |  |
| HOMA-IR decreased to normal ^c^ | 4.15 (0.50-34.24) | 0.19 |  |  |  |  | - | 0.99 |  |  |  |

^a^ Hypertension was defined as systolic blood pressure (SBP) ≥ 140 mmHg or diastolic blood pressure (DBP) ≥ 90 mmHg; ^b^ Multivariable analysis would be further adjusted by smoke, and the variate of smoke was categorized according to the number of cigarettes as 0 (never smoker), 1-9, 10-19, 20-39 and over 40; ^c^ GGT decreased to normal was defined as GGT level ≤ 50 U/L; UA decreased to normal was defined as uric acid level ≤ 420 umol/L for male and ≤ 360 umol/L for female; CHOL decreased to normal was defined as CHOL level ≤ 5.7 mmol/L; TG decreased to normal was defined as TG level ≤ 1.7 mmol/L; LDL-C decreased to normal was defined as LDL-C level ≤ 3.4 mmol/L; FBG decreased to normal was defined as FBG level ≤ 6 mmol/L; HOMA-IR decreased to normal was defined as HOMA-IR level ≤ 2.69.


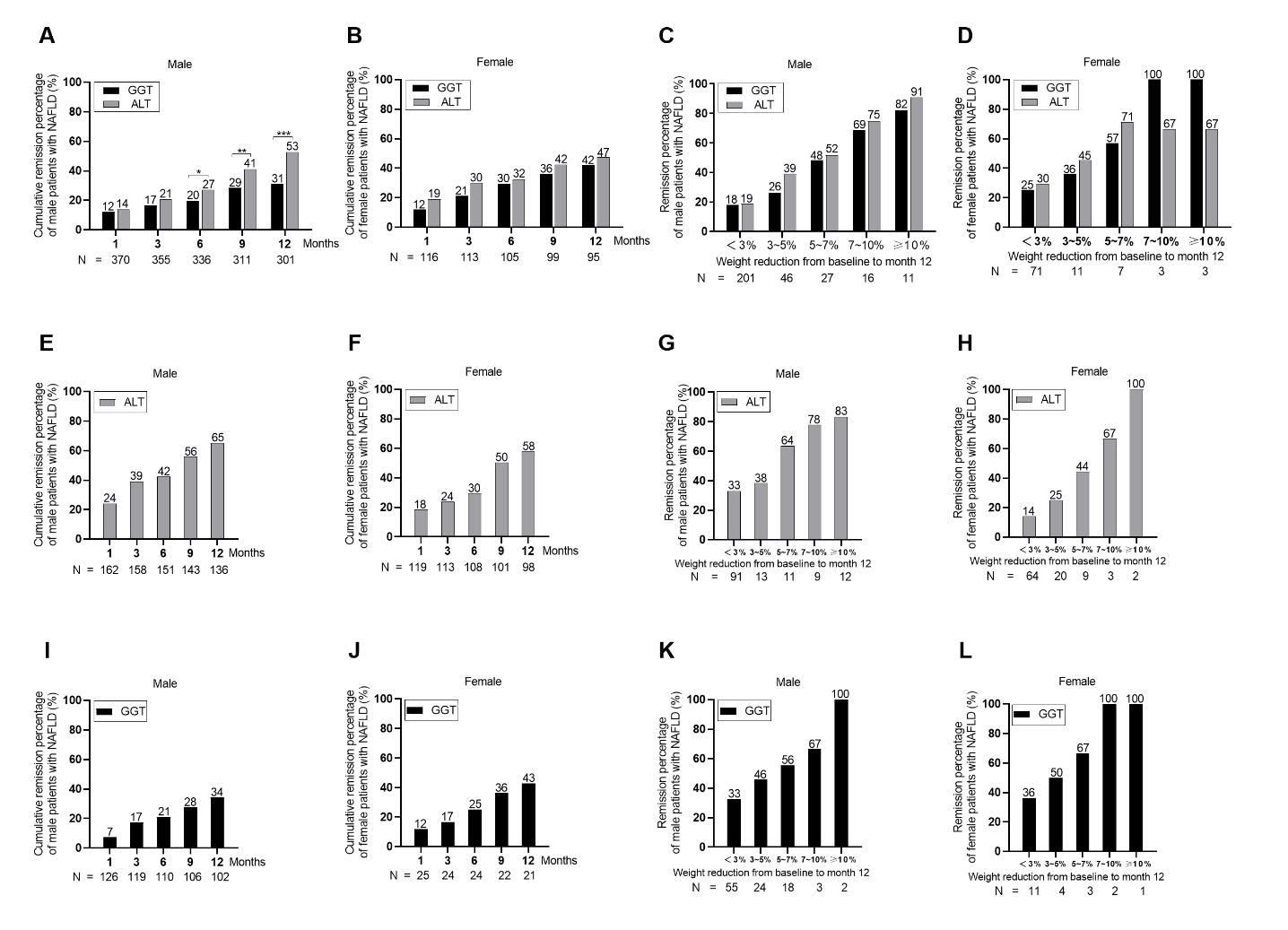


**Additional file 2.** Cumulative normalization rates of ALT and GGT levels after 12 months of treatment. Cumulative normalization rates of ALT or GGT levels in the groups of **(A)** male patients and **(B)** female patients with abnormal levels of both ALT and GGT; **(E)** the male group and **(F)** the female group with abnormal ALT levels only; and **(I)** the male group and **(J)** the female group with abnormal GGT levels only. Relationships between the weight change ratio and ALT or GGT normalization rates **(C)** in the male group and **(D)** the female group with abnormal levels of both ALT and GGT; **(G)** the male group and **(H)** the female group with abnormal ALT levels only; and **(K)** the male group and **(L)** the female group with abnormal GGT levels only.


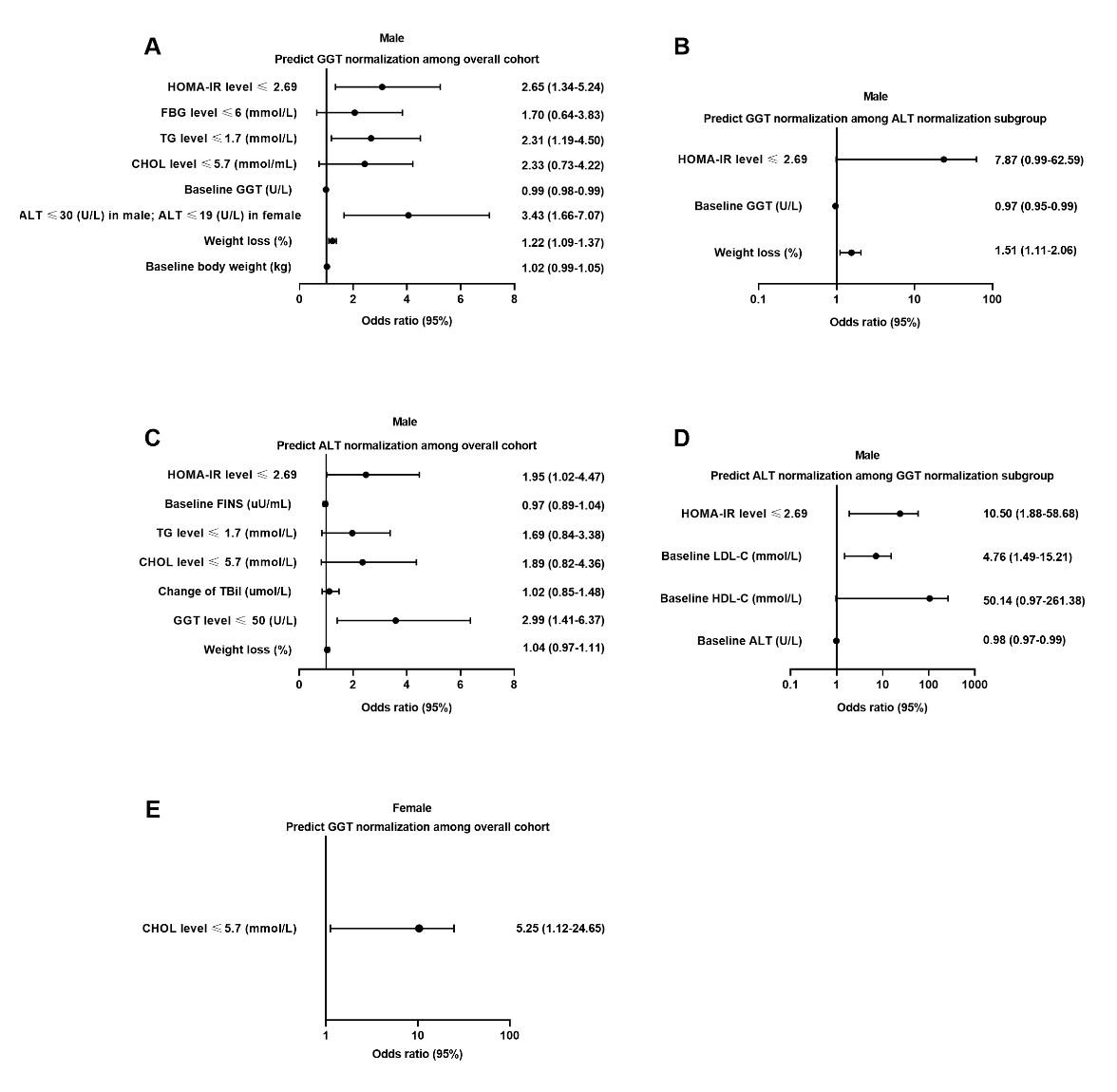


**Additional file 3.** Forest plot predicting the normalization of GGT and ALT. Prediction of GGT normalization in all male populations **(A)** and the normal ALT male population **(B)**; prediction of ALT normalization in all male populations **(C)** and the normal GGT male population **(D)**; prediction of GGT normalization in all female populations **(E)**.


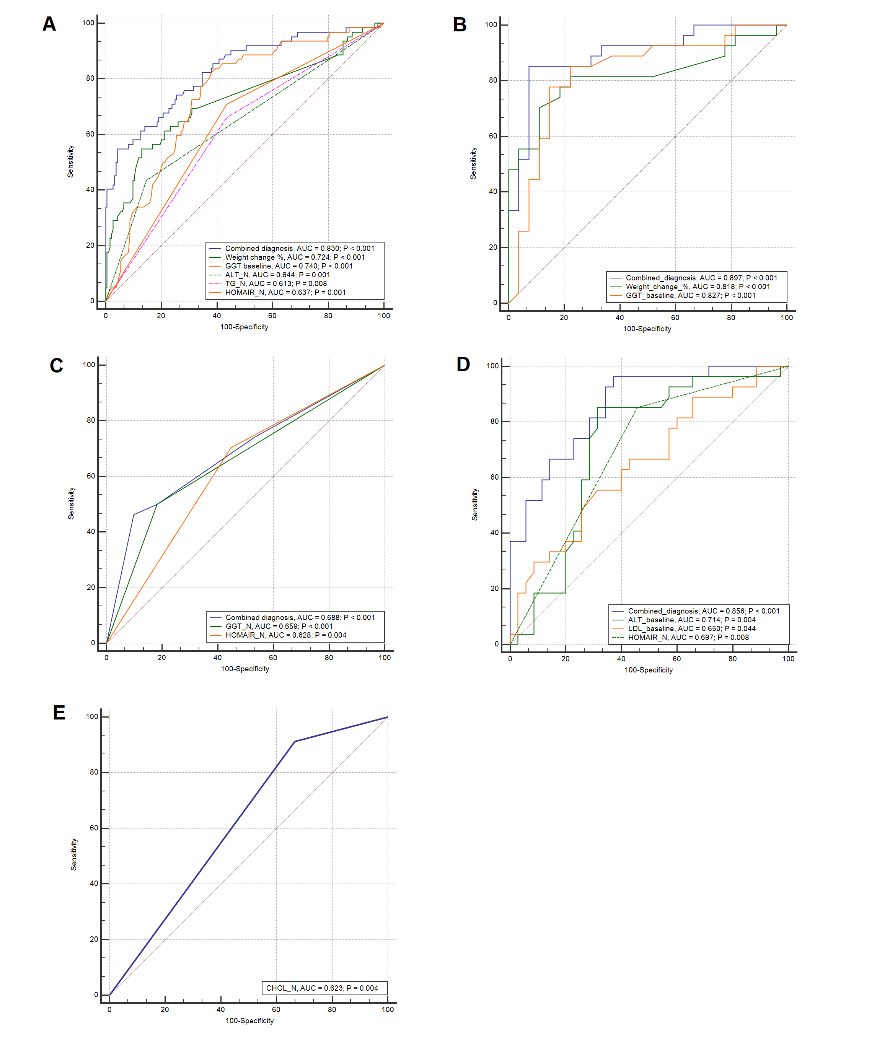


**Additional file 4.** ROC curves predicting the normalization of GGT and ALT. Prediction of GGT normalization in all male populations **(A)** and the normal ALT male population **(B)**; prediction of ALT normalization in all male populations **(C)** and the normal GGT male population **(D)**; prediction of GGT normalization in all female populations **(E)**.


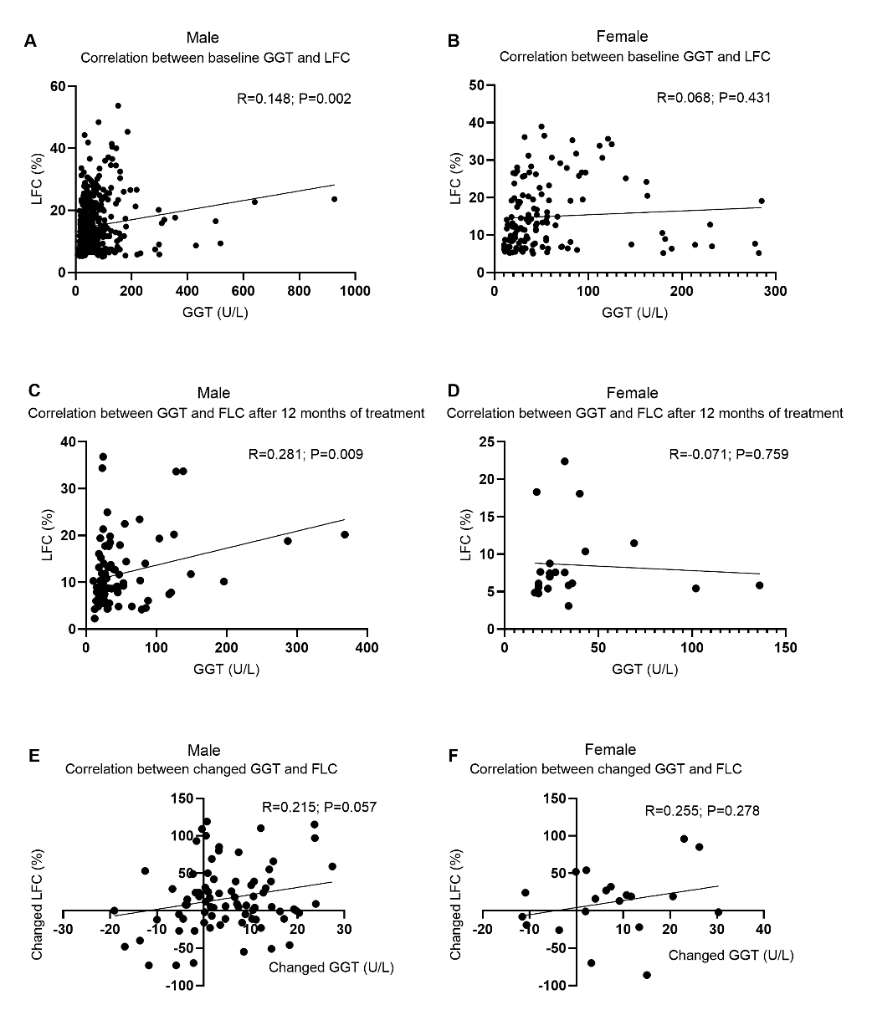


**Additional file 5.** Correlations between liver fat content determined using MRI-PDFF and GGT levels in 630 patients (461 male and 169 female) with NAFLD. Scatter plots of the correlations between GGT levels and the liver fat content in male patients **(A)** at baseline and **(C)** after 12 months of treatment and **(E)** the changes from baseline to month 12; and in female patients **(B)** at baseline and **(D)** after 12 months of treatment and **(F)** the changes from baseline to month 12.


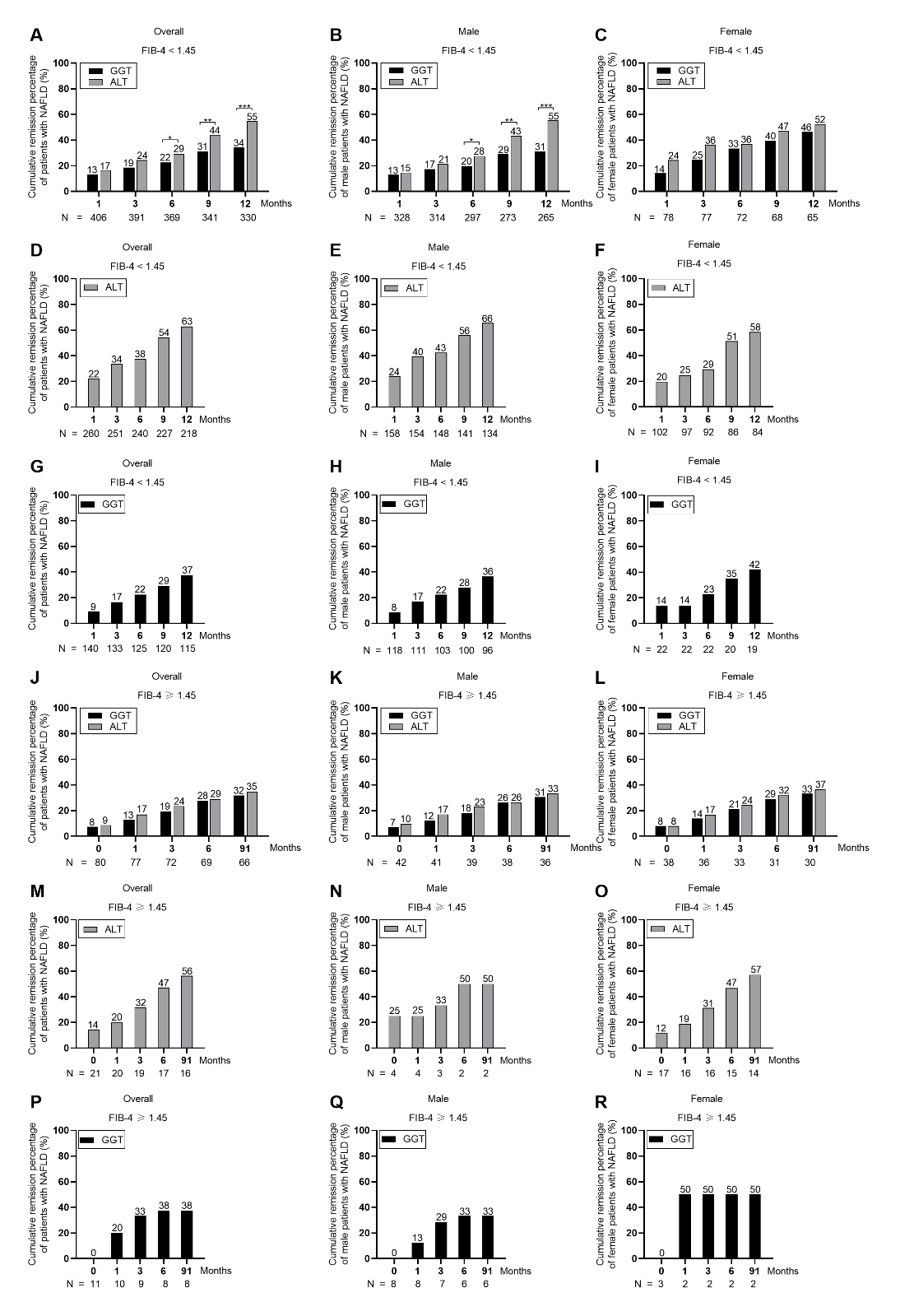


**Additional file 6.** Cumulative normalization rates of ALT and GGT levels in patients with normal and abnormal baseline FIB-4 index after 12 months of treatment. Cumulative normalization rates of ALT or GGT levels in **(A)** overall patients, **(B)** the male group and **(C)** the female group of normal baseline FIB-4 index with abnormal levels of both ALT and GGT; in **(D)** overall patients, **(E)** the male group and **(F)** the female group with abnormal ALT levels only; and in **(G)** overall patients, **(H)** the male group and **(I)** the female group with abnormal GGT levels only. Cumulative normalization rates of ALT or GGT levels in **(J)** overall patients, **(K)** the male group and **(L)** the female group of abnormal baseline FIB-4 index with abnormal levels of both ALT and GGT; in **(M)** overall patients, **(N)** the male group and **(O)** the female group with abnormal ALT levels only; and in **(P)** overall patients, **(Q)** the male group and **(R)** the female group with abnormal GGT levels only.


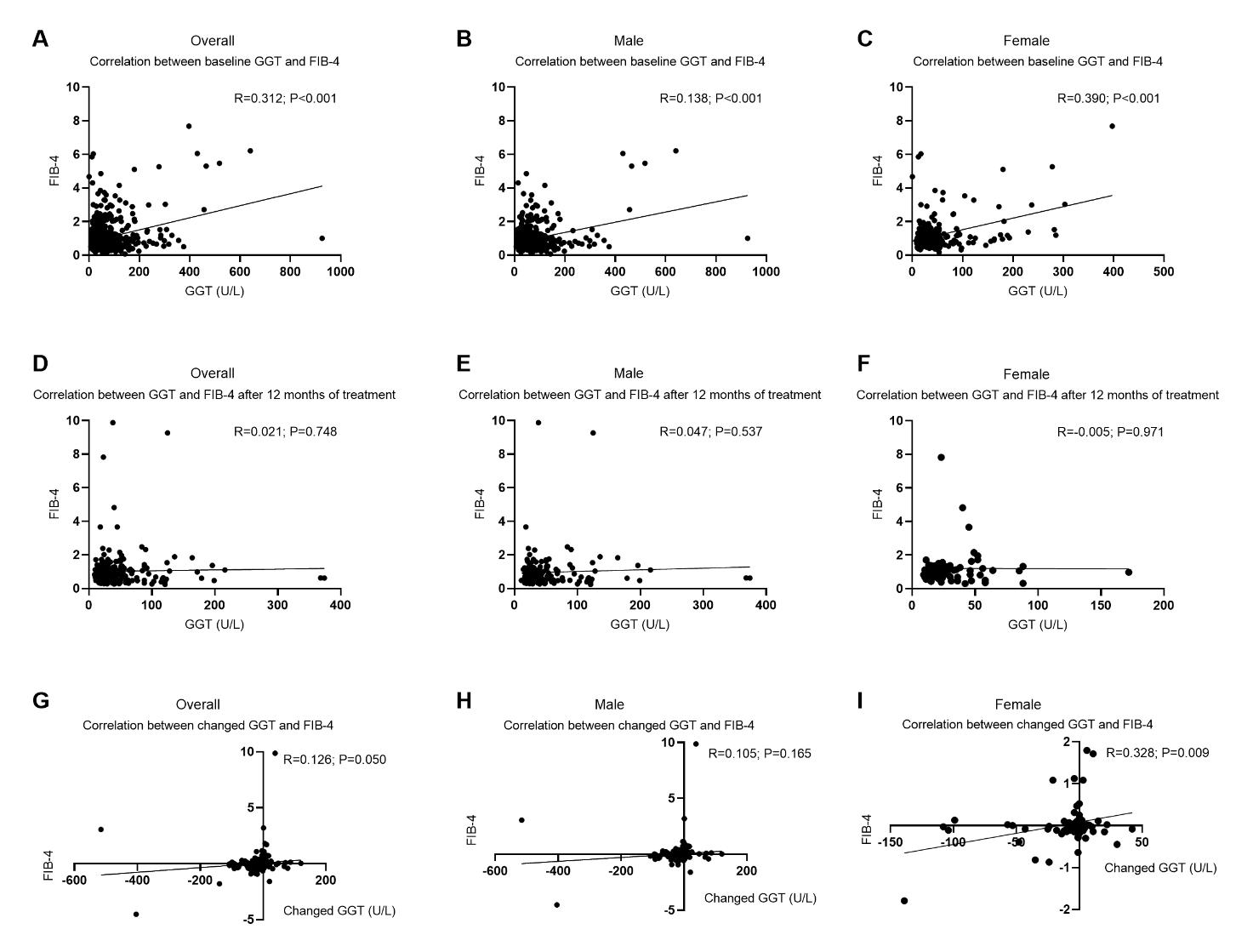


**Additional file 7.** Correlations between GGT levels and the FIB-4 index. Scatter plots of the correlations between GGT levels and the FIB-4 index in **(A)** overall patients, **(B)** male patients and **(C)** female patients. Scatter plots of the correlations between GGT levels and FIB-4 index after 12 months of treatment in **(D)** overall patients, **(E)** male patients and **(F)** female patients. Scatter plots of the correlations between changed GGT levels and changed FIB-4 index after 12 months of treatment in **(G)** overall patients, **(H)** male patients and **(I)** female patients.
