# Supplementary figures and images for: Normalization of γ-glutamyl transferase levels is associated with better metabolic control in individuals with nonalcoholic fatty liver disease
Source: BMC Gastroenterol. 2021 May 10;21:215. doi: 10.1186/s12876-021-01790-w (PMC8112063; doi:10.1186/s12876-021-01790-w)

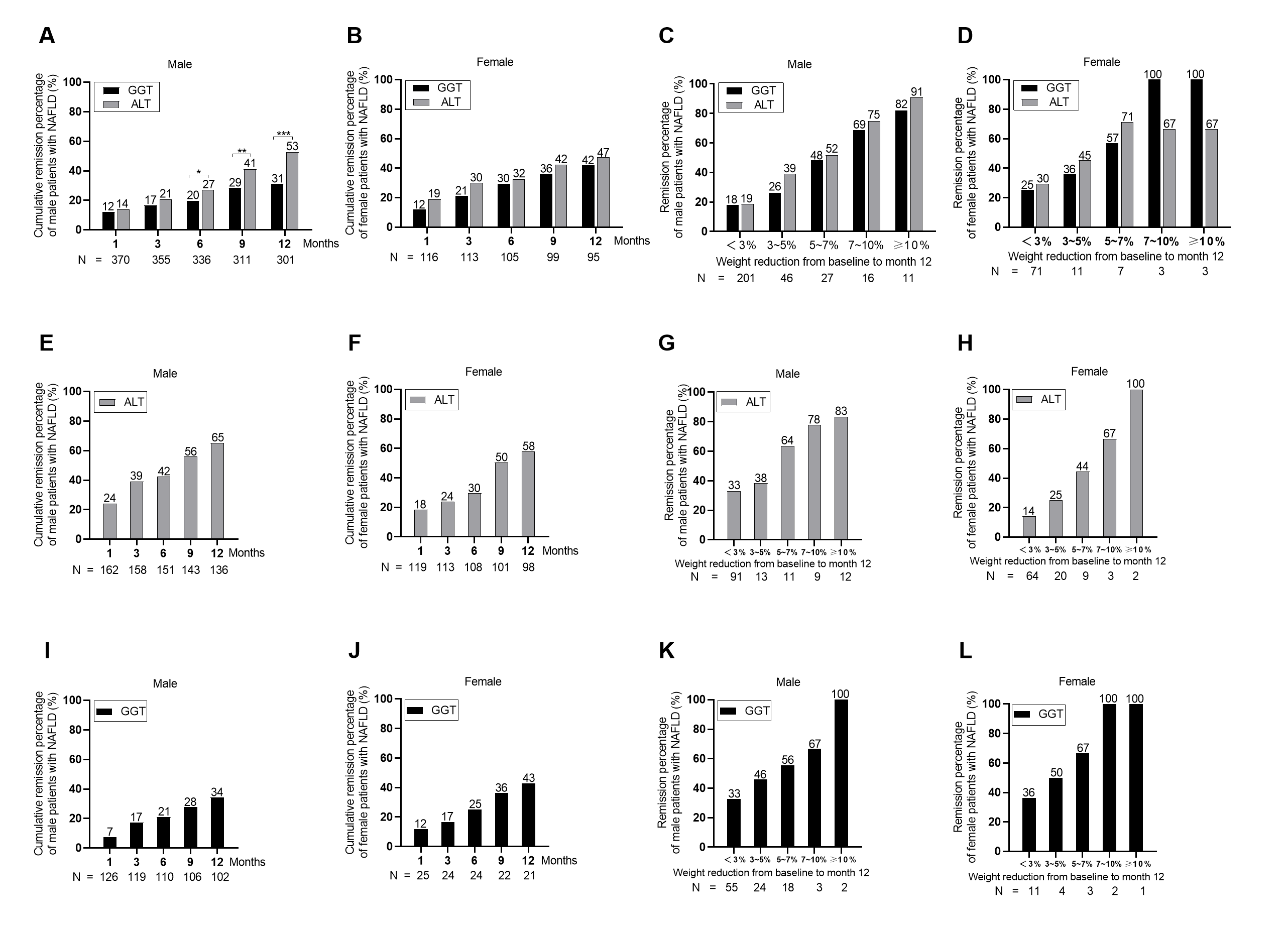

Supplement: Supplementary file 2 — Additional file 2. Cumulative normalization rates of ALT and GGT levels after 12 months of treatment. Cumulative normalization rates of ALT or GGT levels in the groups of (a) male patients and (b) female patients with abnormal levels of both ALT and GGT; (e) the male group and (f) the female group with abnormal ALT levels only; and (i) the male group and (j) the female group with abnormal GGT levels only. Relationships between the weight change ratio and ALT or GGT normalization rates (c) in the male group and (d) the female group with abnormal levels of both ALT and GGT; (g) the male group and (h) the female group with abnormal ALT levels only; and (k) the male group and (l) the female group with abnormal GGT levels only. [file 12876_2021_1790_MOESM2_ESM.tif]

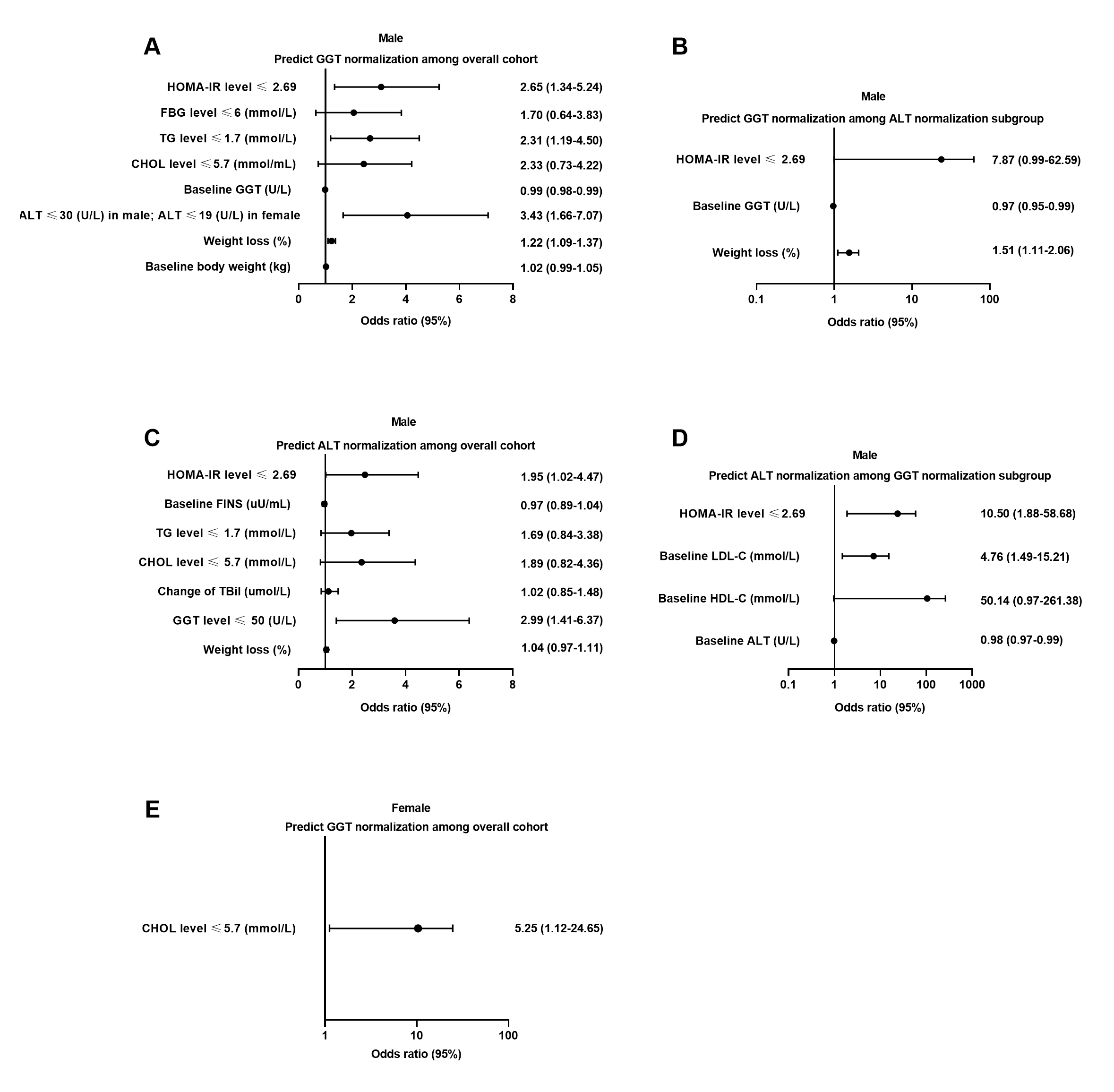

Supplement: Supplementary file 3 — Additional file 3. Forest plot predicting the normalization of GGT and ALT. Prediction of GGT normalization in all male populations (a) and the normal ALT male population (b); prediction of ALT normalization in all male populations (c) and the normal GGT male population (d); prediction of GGT normalization in all female populations (e). [file 12876_2021_1790_MOESM3_ESM.tif]

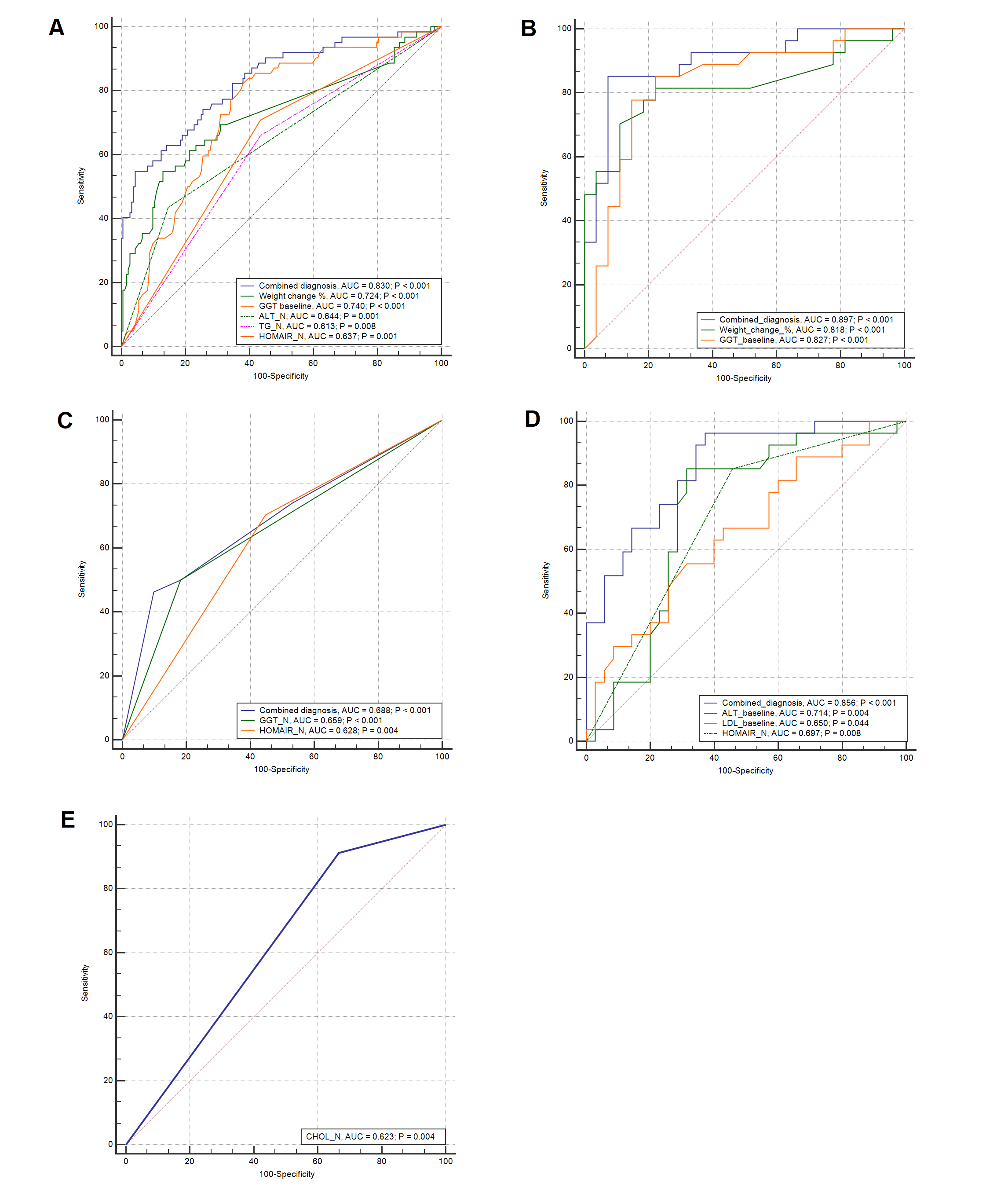

Supplement: Supplementary file 4 — Additional file 4. ROC curves predicting the normalization of GGT and ALT. Prediction of GGT normalization in all male populations (a) and the normal ALT male population (b); prediction of ALT normalization in all male populations (c) and the normal GGT male population (d); prediction of GGT normalization in all female populations (e). [file 12876_2021_1790_MOESM4_ESM.tif]

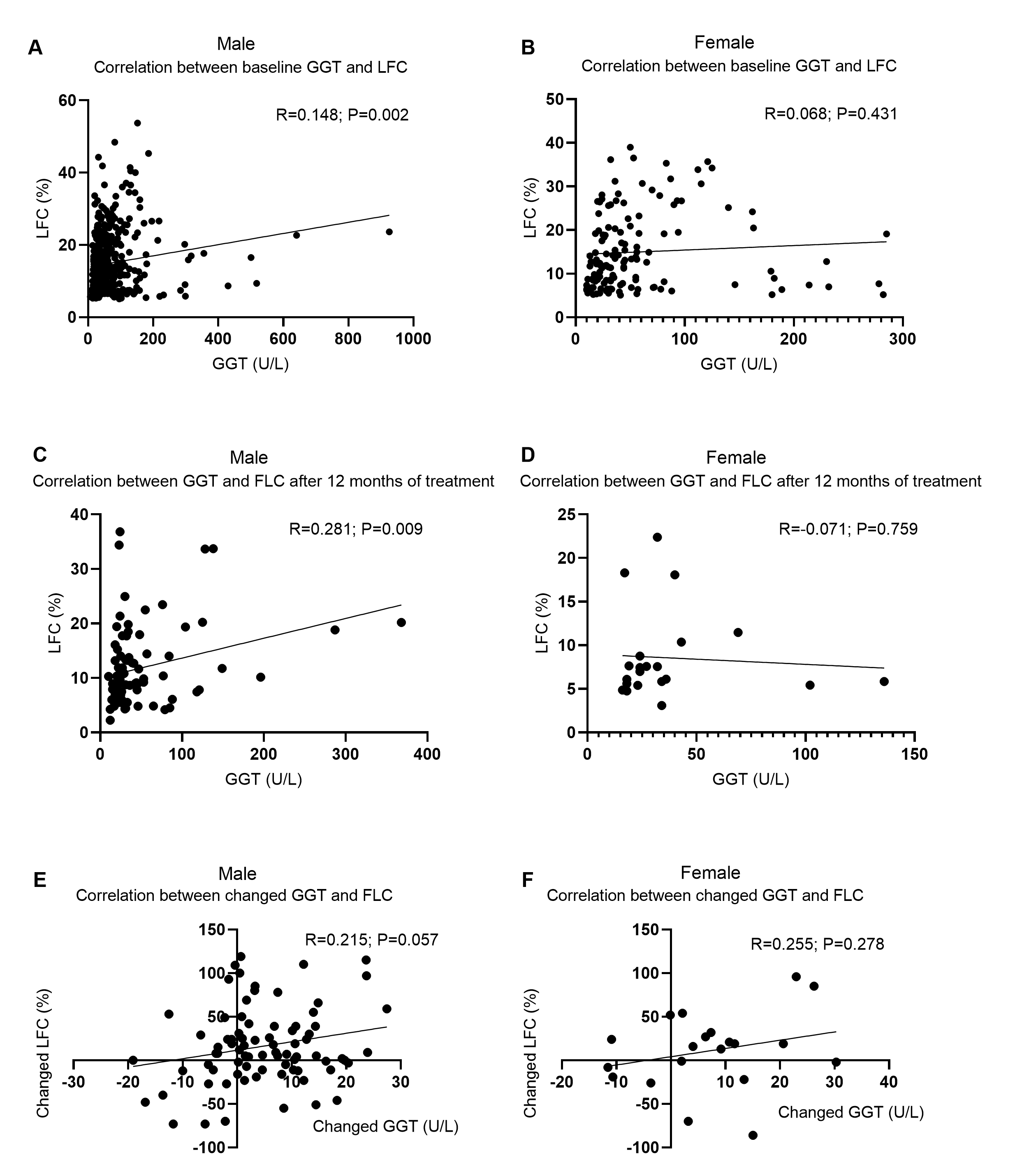

Supplement: Supplementary file 5 — Additional file 5. Correlations between liver fat content determined using MRI-PDFF and GGT levels in 630 patients (461 male and 169 female) with NAFLD. Scatter plots of the correlations between GGT levels and the liver fat content in male patients (a) at baseline and (c) after 12 months of treatment and (e) the changes from baseline to month 12; and in female patients (b) at baseline and (d) after 12 months of treatment and (f) the changes from baseline to month 12. [file 12876_2021_1790_MOESM5_ESM.tif]

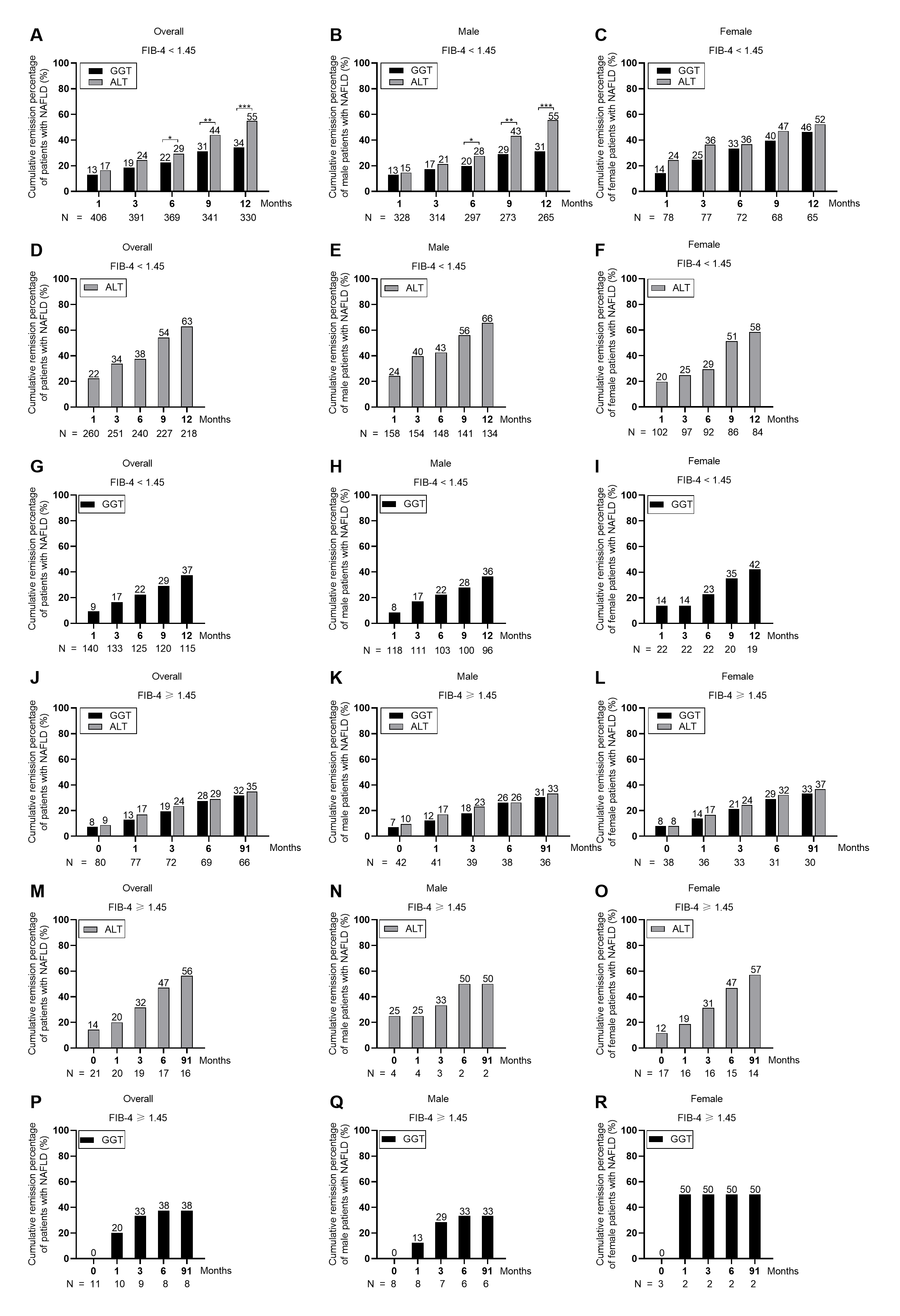

Supplement: Supplementary file 6 — Additional file 6. Cumulative normalization rates of ALT and GGT levels in patients with normal and abnormal baseline FIB-4 index after 12 months of treatment. Cumulative normalization rates of ALT or GGT levels in (a) overall patients, (b) the male group and (c) the female group of normal baseline FIB-4 index with abnormal levels of both ALT and GGT; in (d) overall patients, (e) the male group and (f) the female group with abnormal ALT levels only; and in (g) overall patients, (h) the male group and (i) the female group with abnormal GGT levels only. Cumulative normalization rates of ALT or GGT levels in (j) overall patients, (k) the male group and (l) the female group of abnormal baseline FIB-4 index with abnormal levels of both ALT and GGT; in (m) overall patients, (n) the male group and (o) the female group with abnormal ALT levels only; and in (p) overall patients, (q) the male group and (r) the female group with abnormal GGT levels only. [file 12876_2021_1790_MOESM6_ESM.tif]

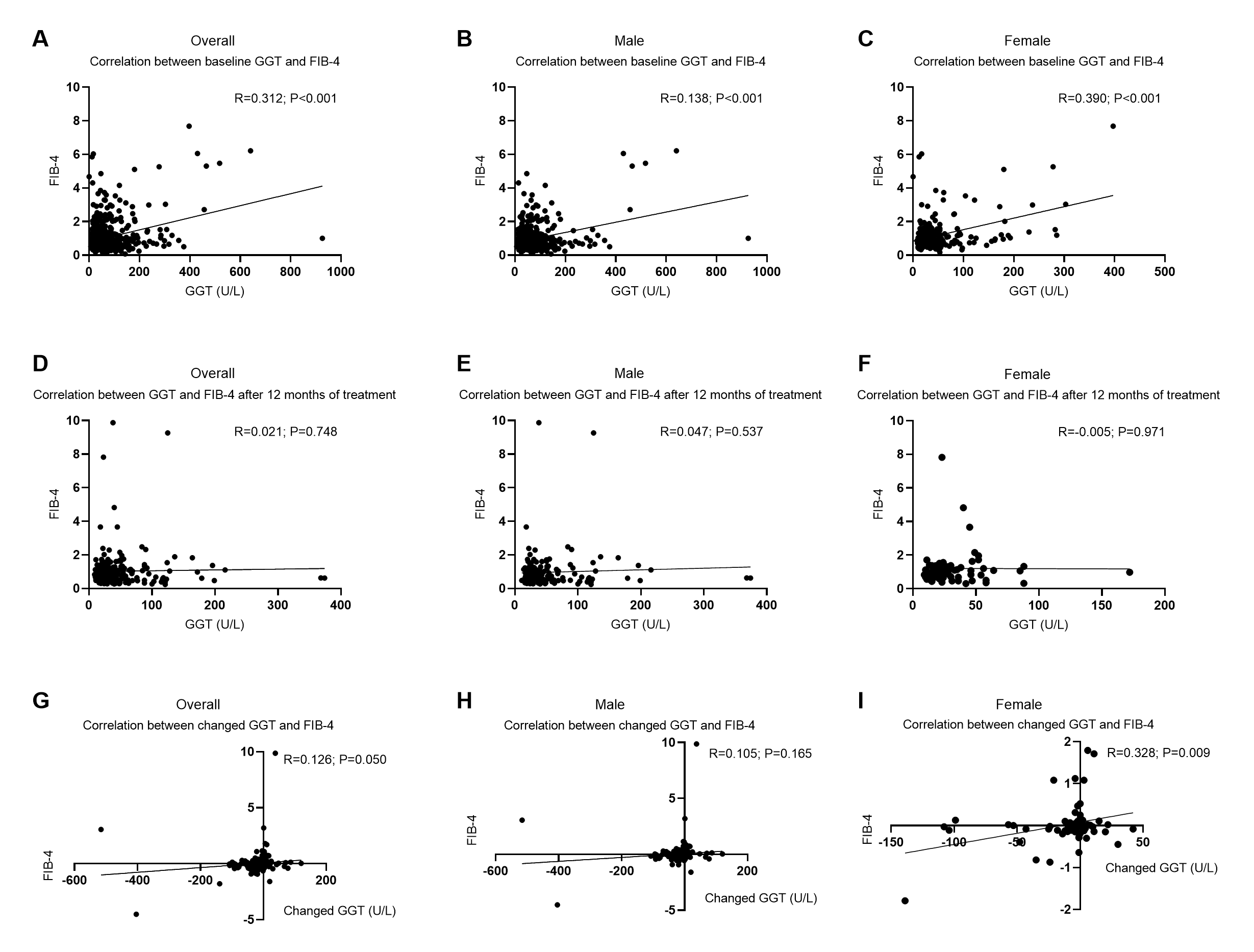

Supplement: Supplementary file 7 — Additional file 7. Correlations between GGT levels and the FIB-4 index. Scatter plots of the correlations between GGT levels and the FIB-4 index in (a) overall patients, (b) male patients and (c) female patients. Scatter plots of the correlations between GGT levels and FIB-4 index after 12 months of treatment in (d) overall patients, (e) male patients and (f) female patients. Scatter plots of the correlations between changed GGT levels and changed FIB-4 index after 12 months of treatment in (g) overall patients, (h) male patients and (i) female patients. [file 12876_2021_1790_MOESM7_ESM.tif]
